# Neoadjuvant toripalimab plus axitinib for clear cell renal cell carcinoma with inferior vena cava tumor thrombus: NEOTAX, a phase 2 study

**DOI:** 10.1038/s41392-024-01990-2

**Published:** 2024-10-04

**Authors:** Liangyou Gu, Cheng Peng, Qiyang Liang, Qingbo Huang, Deqiang Lv, Houming Zhao, Qi Zhang, Yu Zhang, Peng Zhang, Shichao Li, Junnan Xu, Luyao Chen, Yongpeng Xie, Jinhang Li, Gang Guo, Xu Zhang, Baojun Wang, Xin Ma

**Affiliations:** 1 Department of Urology, Chinese PLA General Hospital, Beijing, China; 2 Chinese PLA Medical School, Beijing, China; 3 China National Center for Bioinformation, Beijing, China; 4 Beijing Institute of Genomics, Chinese Academy of Sciences, Beijing, China; 5 University of Chinese Academy of Sciences, Beijing, China; 6 Department of Urology, The First Affiliated Hospital of Nanchang University, Nanchang, China; 7 Department of Urology, The First Affiliated Hospital of Chongqing Medical University, Chongqing, China; 8 Department of Pathology, Chinese PLA General Hospital, Beijing, China

**Keywords:** Urological cancer, Urological cancer

## Abstract

The potential benefit of neoadjuvant toripalimab plus axitinib in cases with clear cell renal cell carcinoma (ccRCC) and inferior vena cava tumor thrombus (IVC-TT) remains unclear. NEOTAX was a phase 2 study to investigate the efficacy and safety of neoadjuvant toripalimab plus axitinib in patients with ccRCC and IVC-TT (ChiCTR2000030405). The primary endpoint was the down-staging rate of IVC-TT level. Secondary endpoints included change in TT length, response rate, percentage change in surgical approach, surgical morbidity, progression-free survival (PFS), safety, and biomarker analyses. In all, 25 patients received study treatment, 44.0% (11/25) patients had a reduction in thrombus level, and none experienced an increase in Mayo level. The median change in tumor thrombus length was −2.3 cm (range: −7.1 to 1.1 cm). Overall, 61.9% (13/21) patients experienced changes in surgical strategy compared with planned surgery, three patients experienced major complications. The median PFS was 25.3 months (95% CI: 17.0-NE). The 1-year PFS was 89.1% (95% CI: 62.7–97.2). No any of grade 4 or 5 treatment-related adverse event was identified. Biopsy samples of non-responders exhibited increased T cytotoxic cell infiltration, but these cells were predominantly PD-1 positive. Biopsy samples of responders exhibited lower T helper cells, however, their subtype, regulatory T cells remained unchanged. In surgical samples of the TT, non-responders exhibited increased CD8T_01_GZMK_CXCR4 subset T cells. NEOTAX met preset endpoints proving that toripalimab in combination with axitinib downstages IVC-TT in a significant proportion of patients leading to simplification in the procedure of surgery.

## Introduction

Renal cell carcinoma (RCC) has a biologic propensity for vascular invasion leading to renal vein or inferior vena cava (IVC) tumor thrombus (TT) in 4–15% of cases, which result in poor survival.^1^ Taking into the height of TT, it can be classified to level 0–V based on Mayo Clinic classification system.^2^ The current standard of care is radical nephrectomy accompanying IVC thrombectomy for localized RCC with venous tumor thrombus. However, the procedures during surgery, especially TT resection and IVC reconstruction, are associated with high rate of surgical morbidity and mortality.^3^ Moreover, surgical complexity and rate of complication increase with the level of TT,^1,2^ the postoperative complications have been described to occur in up to 22.9–45% of cases with level III/IV TT.^2,4^ It is important to control morbidity and mortality by downstaging TT levels and simplifying surgical procedures.

Therefore, neoadjuvant therapy with systematic agents to reduce the height of inferior vena cava tumor thrombus (IVC-TT) has aroused researchers’ interest.^5,6^ Karakiewicz et al.^7^ from Canada initially reported a patient with RCC and right atrial thrombus who refused the sternotomy. After two cycles of neoadjuvant sunitinib treatment, the TT was down-staged from level IV to II, and surgical coverage was significantly reduced. After that, many centers have applied pre-surgical tyrosine kinase inhibitors (TKIs) in non-metastatic or metastatic RCC with tumor thrombus.^8,9^ Although tumor thrombus volume and height were often reduced, only about 30% of patients achieve a reduced stage at TT level.^5,9^

To date, there is no standard neoadjuvant therapy which is recommended for improve surgical safety or overall survival in locally advanced RCC.^10^ Due to the advantages of immunotherapy-based combinations (IBCs) versus sunitinib, IBCs became the first-line treatments that were recommended for advanced RCC.^3^ Taken that IBCs has a higher objective response rate (ORR) than TKIs, which can be a better choice for neoadjuvant therapy. In 2019, Labbate et al.^11^ described a case of RCC with level IV TT achieving pathologic complete response in TT after neoadjuvant nivolumab and ipilimumab. The following case report and series have also confirmed the safety and feasibility of neoadjuvant IBCs in RCC with TT.^8,12^ However, the level II evidence for this issue is lacking.

RENOTORCH is a phase 3 trial that assessed the efficacy of the combination toripalimab and axitinib in metastatic clear cell RCC (ccRCC) compared to sunitinib, the combination demonstrated an ORR of 56.7% compared to 30.8% in the sunitinib arm.^13^ We hypothesized that neoadjuvant toripalimab combined with axitinib can achieve better down-staging rate of IVC-TT level than TKI alone. This phase 2 study (NEOTAX) assessed the efficacy and safety of neoadjuvant toripalimab in combination with axitinib for RCC with IVC-TT. Considering that axitinib has a shorter half-life, fast-acting response and potential immune-activating effects, it has become an ideal presurgical agent of efficacy and utility of neoadjuvant therapy, especially in the setting of IBC treatment.

## Results

### Patient characteristics

**Fig. 1:**
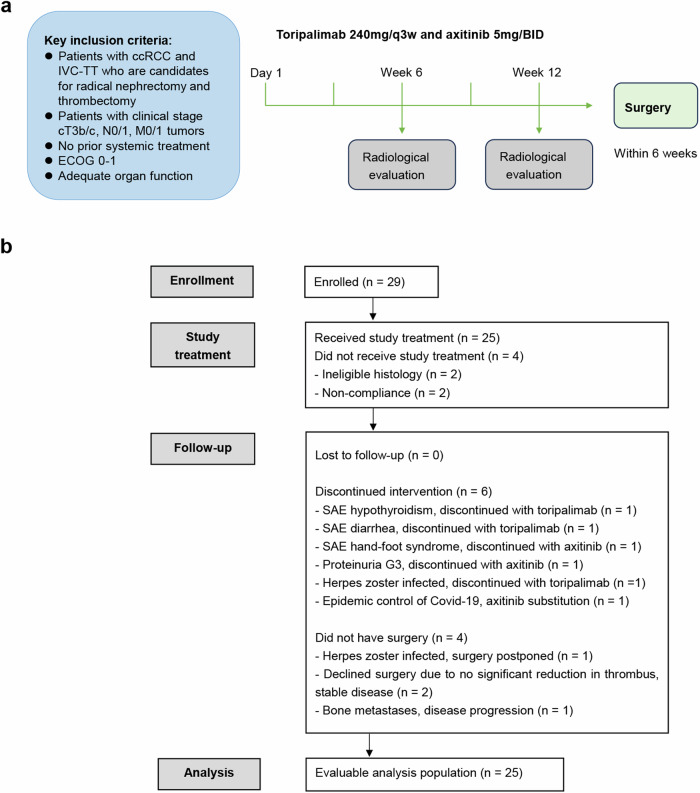
Trial design and conduct. **a** Study schema; **b** CONSORT diagram. Participants who had at least one dose of the study drug were included in the evaluable population, regardless of whether they had surgery

From Mar 2020 to Oct 2023, a total of 29 patients were enrolled in our single center. After baseline biopsy, 2 patients had ineligible histology, 2 patients were identified to be non-compliant, 25 patients received study treatment (Fig. 1b). Baseline demographics and disease characteristics of 25 patients were shown in Table 1. The median age was 58 years (interquartile range [IQR], 51.5–67.5 years), and most were male (*n* = 19, 76.0%). There were 10 (40.0%) patients with lymph node metastasis, 5 (25.0%) patients with distant metastasis. There were 9 (36.0%), 7 (28.0%), 9 (36.0%) patients with Mayo level II, III, IV tumor thrombus, respectively.

**Table 1:** Patient characteristics

Variable	*N* = 25
Median age, year (IQR)	58 (51.5–67.5)
Sex, *n* (%)	
Male	19 (76.0)
Female	6 (24.0)
Median BMI, kg/m^2^ (IQR)	24.1 (21.5–26.2)
ECOG performance status, *n* (%)	
0	22 (88.0)
1	3 (12.0)
Tumor laterality, *n* (%)	
Right	17 (68.0)
Left	8 (32.0)
Clinical T stage, *n* (%)	
T3b	14 (56.0)
T3c	9 (36.0)
T4	2 (8.0)
Clinical N stage, *n* (%)	
N0	15 (60.0)
N1	10 (40.0)
Clinical M stage, *n* (%)	
M0	20 (75.0)
M1	5 (25.0)
Primary tumor size, cm (IQR)	8.2 (7.0–10.6)
Length of the tumor thrombus, cm (IQR)	9.5 (7.1–12.3)
Histological subtype on baseline biopsy, *n* (%)	
ccRCC	25 (100.0)
Mayo level of TT on baseline imaging	
I	0 (0.0)
II	9 (36.0)
III	7 (28.0)
IV	9 (36.0)

*IQR* interquartile range, *BMI* body mass index, *ccRCC* clear cell renal cell carcinoma, *TT* tumor thrombus

### Radiological efficacy

**Fig. 2:**
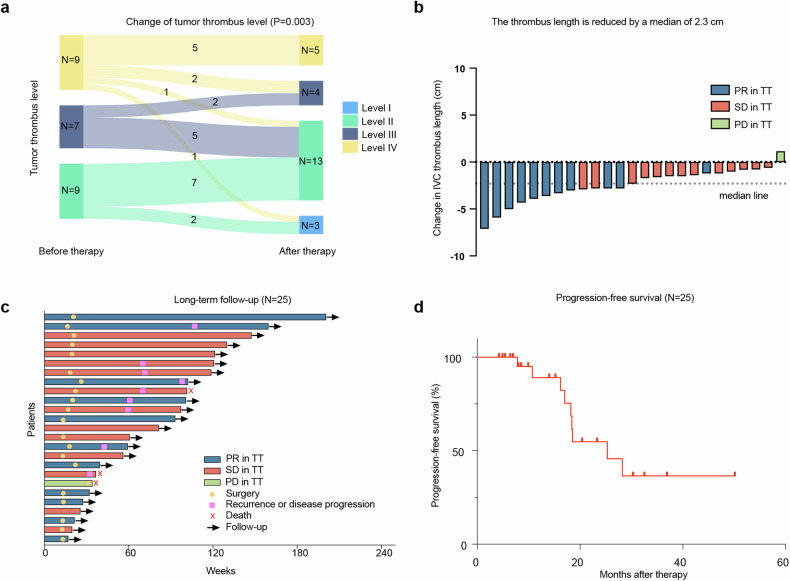
Treatment response. **a** Sankey diagram showing number of patients with tumor thrombus level II–IV and their changes between pre- and post-treatment; **b** Waterfall plot of the best change in tumor thrombus length; **c** Swimmer plot of all 25 patients; **d** Progression-free survival. PR partial response, SD stable disease, PD progressive disease

In the first stage, six (37.5%) of 16 evaluable patients initially enrolled achieved a reduction in thrombus level, which met the statistical criteria to proceed to the stage II enrollment. After 12 weeks treatment, among the 25 eligible and evaluable patients finally enrolled, 44.0% (11/25) patients experienced a reduction in TT level (IV–III in two patients, IV–II in one patient, IV–I in one patient, III–II in five patients, and II–I in two patients). Neoadjuvant therapy significantly reduced the Mayo level of patients with tumor thrombus (Std. MH Statistic = 2.985, *P* = 0.003). The remaining 14 patients were stable in thrombus level, and none experienced an increase in Mayo level (Fig. 2a).

Though 56.0% (14/25) patients were stable in thrombus level, 13 of them had a reduction in TT length, therefore 24/25 (96.0%) patients had varying degrees of TT length reduction (range 5–79%). Only one patient (4.0%) experienced an increase in TT length with 20.7% (absolute value 1.1 cm). The median change in TT length was −2.3 cm (range: −7.1 to 1.1 cm) (Fig. 2b).

According to the RECIST criteria, the best overall tumor response was 40.0% (10/25) patients with partial response (PR) and 60.0% (15/25) patients with stable disease. After 12 weeks of neoadjuvant therapy, 11 of 25 patients (44.0%) achieved a PR in IVC-TT (>30% shrinkage in tumor thrombus length), and one patient (4.0%) developed progressive disease in TT without increased Mayo level.

### Change of surgical approach

Twenty-one patients suitable for surgery underwent radical nephrectomy accompanying IVC thrombectomy, with four cases did not have surgery as depicted in Fig. 1b. One patient had a surgery delay due to herpes zoster infection, although the size of the primary tumor and tumor thrombus was significantly reduced on radiography. Considering the high risk of major surgical complications and limited response in TT (level IV, no significant shrinkage of the intra-atrial tumor thrombus), two patients declined surgery. Additionally, one patient infected with herpes zoster subsequently developed bone metastases, leading to the loss of the opportunity for surgical intervention.

Moreover, 61.9% (13/21) patients experienced changes in surgical strategy compared with planned surgery. Four patients with initial level IV TT avoided incision of right atrium and cardiopulmonary bypass, which due to downgrading of Mayo level (two patients from IV to III, one patient from IV to II, one patient from IV to I). Although the thrombi remain stable in Mayo level IV, one patient underwent transabdominal-transdiaphragmatic robotic IVC thrombectomy without cardiopulmonary bypass, because the proximal of thrombi shrink back into intrapericardial IVC from the right atrium after treatment. Four patients with initial level III thrombus were planned to maximum extent of liver mobilization, the first porta hepatis occlusion and the supra-hepatic IVC incision, neither of these manoeuvers was needed due to downgrading. Four patients with initial level II thrombus had less extensive liver mobilization with thrombi shrink back from retro-hepatic vena cava to infra-hepatic vena cava, but three of them had no change in Mayo level. One patient did not change surgical plan with downstage of Mayo level from II to I, regarding to the extent of liver mobilization and IVC clamping type. The surgery was successfully completed as scheduled for the remaining eight patients (Supplementary Fig. S1).

### Perioperative outcomes

Twenty-one patients successfully underwent radical nephrectomy accompanying IVC thrombectomy. Table 2 presented the perioperative outcomes. The median operative time was 314 min (IQR: 202.5–512.5 min), while the median estimated blood loss was 800 mL (IQR: 300–2000 ml). Twelve patients (57.1%) required intro-operative blood transfusion, with a median volume of 540 ml (range: 0–3715 ml). Eleven patients (52.4%) were transferred to the intensive care unit after operation, with a median stay of 2 days.

**Table 2:** Perioperative outcomes

Variable	*N* = 21
Surgical approach, *n* (%)	
Open	4 (19.0)
Robotic	17 (81.0)
Surgery type, *n* (%)	
Thrombectomy	15 (71.4)
Cavectomy	6 (28.6)
Operative time (min), median (IQR)	314 (202.5–512.5)
Estimated blood loss (ml), median (IQR)	800 (300–2000)
Intra-operative blood transfusion	
No. pts (%)	12 (57.1)
Median ml (range)	540 (0–3715)
ICU, *n* (%)	11 (52.4)
ICU stay (d), median (IQR)	2 (0–3)
Median days (IQR)	
To surgical drain removal	4 (3–7)
To full ambulation	1 (1–3)
To oral feeding	2 (1–3)
Postoperative hospital stay (d), median (IQR)	8 (6–11)
Conversion to open, *n* (%)	0 (0.0)
Postoperative complications, *n* (%)	
Grade I	2 (9.5)
Grade II	7 (33.3)
Grade III	0 (0.0)
Grade IV	2 (9.5)
Grade V	1 (4.8)
Changes in surgical strategy, *n* (%)	13 (61.9)
Incision of right atrium avoided	5 (23.8)
Cardiopulmonary bypass avoided	5 (23.8)
Hepatic artery occlusion avoided	5 (23.8)
Less extensive liver mobilization	9 (42.9)

*IQR* interquartile range, *ICU* intensive care unit

Postoperative complications occurred in 57.1% (12/21) patients. The most common minor complications were transient renal disfunction and the need for a postoperative transfusion. Three patients experienced major complications. One patient experienced renal failure requiring dialysis (Grade IVa). One patient had a cardiorespiratory arrest necessitating cardiopulmonary resuscitation (Grade IVa). One patient died of hemorrhagic shock and disseminated intravascular coagulation due to excessive bleeding during surgery (Grade V, 4.8% mortality rate). None of these major complications were caused by TKI therapy or immunotherapy. Despite being a common concern following IBC therapy, there were no reported complications with wound healing. Adhesions of intra-abdominal tissues have been observed in some patients, however, they did not result in a significant escalation in surgical complexity or necessitate a transition to open surgery.

### Follow-up

The median follow-up time for the whole cohort was 23.3 months (95% confidence interval [CI]: 9.9–29.7). The median progression-free survival (PFS) was 25.3 months (95% CI: 17.0-NE). Tumor progression (tumor recurrence, new metastases appeared, original metastases progressed) occurred in nine patients (36.0%), seven of them progressed postoperatively (Fig. 2c). The 1-year and 2-year PFS was 89.1% (95% CI: 62.7–97.2) and 54.8% (95% CI: 27.5–75.6) (Fig. 2D). There were two deaths (8.0%) reported during follow-up, including one patient who experienced disease progression and one patient who died from COVID-19 (Fig. 2c).

### Safety

Treatment-related AEs (TRAEs) happened in all patients (100.0%) receiving toripalimab in combination with axitinib, and most of them were grade 1 or 2 (Table 3). AEs were in line with former studies, and none TRAEs delay operation. No any of grade 4 or 5 TRAEs was identified. In total, grade 3 TRAEs observed in seven patients (28.0%). Hypertension (8.0%), and proteinuria (8.0%) were the most common grade 3 TRAEs. Two patients discontinued with toripalimab due to grade 3 hypothyroidism and grade 3 diarrhea, respectively. Discontinuation of axitinib occurred in two patients due to grade 3 palmar-plantar erythrodysesthesia (PPE) syndrome and grade 3 proteinuria, respectively. Another patient experienced herpes zoster, leading to the discontinuation of toripalimab and postponement of surgery. Furthermore, one patient suspended axitinib therapy for 2 weeks due to the COVID-19 pandemic quarantine and subsequent lack of access to medication.

**Table 3:** Safety outcomes (any grade ≥10% in the total evaluable population)

	*N* = 25	
	Any grade	Grade 3
Any TRAE, *n* (%)	25 (100.0)	7 (28.0)
TRAEs (≥10%), *n* (%)		
Diarrhea	9 (36.0)	1 (4.0)
Hypertension	9 (36.0)	2 (8.0)
Proteinuria	9 (36.0)	2 (8.0)
Fatigue	8 (32.0)	0
Rash	7 (28.0)	0
Hypertriglyceridemia	7 (28.0)	1 (4.0)
Weight decreased	6 (24.0)	1 (4.0)
AST increased	5 (20.0)	0
Voice alteration	4 (16.0)	0
Decreased appetite	4 (16.0)	0
ALT increased	4 (16.0)	0
PPE syndrome	4 (16.0)	1 (4.0)
LDH increased	4 (16.0)	0
TSH increased	3 (12.0)	0
Hypothyroidism	3 (12.0)	1 (4.0)
Dysphonia	3 (12.0)	0
Mucositis	3 (12.0)	0
Constipation	3 (12.0)	0
Nausea	3 (12.0)	0
Gamma-GT increased	3 (12.0)	0

*AST* aspartate aminotransferase, *ALT* alanine aminotransferase, *PPE* palmar-plantar erythrodysesthesia, *LDH* lactate dehydrogenase, *TSH* thyroid-stimulating hormone, *GT* glutamyltransferase

### Translational analyses

**Fig. 3:**
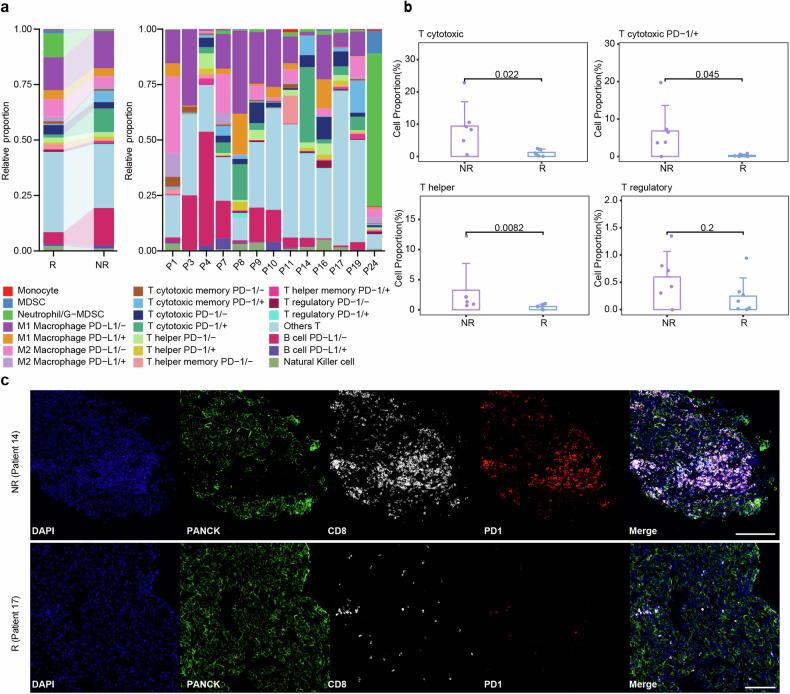
Cell DIVE multiplexed imaging revealed differences in cell composition of biopsy samples from RCC patients before neoadjuvant therapy. **a** Bar chart showed the immune cell composition of RCC patients (*n* = 13) with different TT responses; **b** Bar graphs showed the difference between representative T cells in R (*n* = 7) and NR (*n* = 6) patients; **c** Representative immunofluorescence plots showed the difference in the expression of T cytotoxic PD-1/+ cells between R and NR patients. Scale bars, 100 μm

Preoperative primary tumors puncture samples, available from 18 patients, were investigated for potential molecular biomarkers or immune cell subsets predictive of therapy effectiveness. Thirteen patients were finally included due to detachment or sample quality problems. Our analysis revealed distinct cell types and subtypes (Fig. 3a, Supplementary Table S1). When stratifying patients based on TT response, we observed higher levels of T cytotoxic cells (CD3^+^CD4^−^CD8^+^CD45RO^−^) in non-responders (NR), however, these cells were predominantly PD-1 positive (CD3^+^PD1^+^CD4^−^CD8^+^CD45RO^−^), contributing to an immunosuppressive tumor microenvironment (Fig. 3b, c). Next, we found responders (R) had lower T helper cells (CD3^+^CD4^+^CD8^−^FOXP3^−^CD45RO^−^), however, their subtype, regulatory T cells (CD3^+^CD4^+^CD8^−^FOXP3^+^CD45RO^−^), another major immunosuppressive T-cell population, remained unchanged (Fig. 3b).

**Fig. 4:**
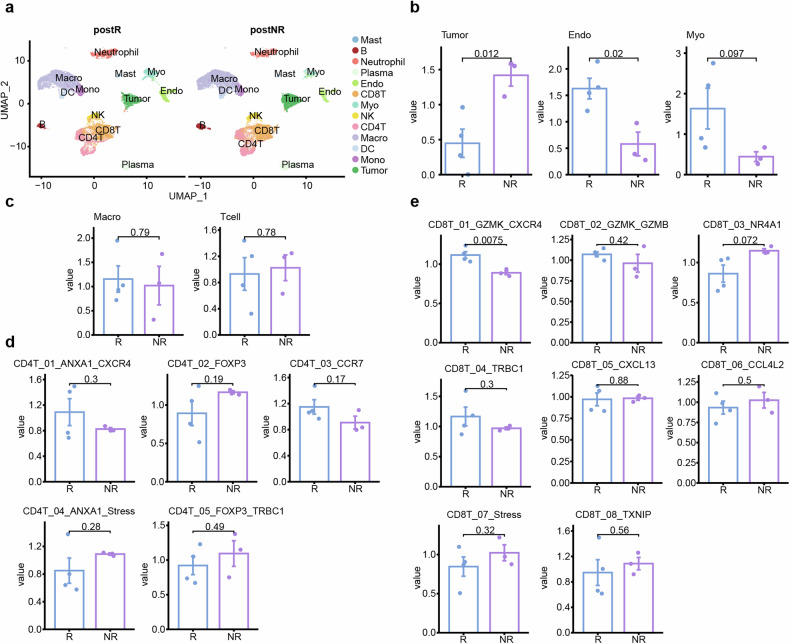
scRNA-seq revealed differences in cell composition of post-treatment samples between R and NR patients. **a** UMAP depicting the distribution of cells between two groups; **b** Bar graphs showed the relative enrichment of major non-immune cells between two groups; **c** Bar graphs showed the relative enrichment of macrophages and T cells in all CD45^+^ cells between two groups; **d** Bar graphs showed the relative enrichment of major CD4^+^T cells’ subtypes between two groups; **e** Bar graphs showed the relative enrichment of major CD8^+^T cells’ subtypes between two groups

To depict the tumor microenvironment, surgical samples of the TT, specifically four from NR patients and three from R patients were used for scRNA-seq. We obtained 41,973 cells following strict quality controls and were broadly categorized into 13 major cell types (Fig. 4a). In the non-immune cells of NR patients, we observed a higher proportion of tumor cells and a lower proportion of endothelial cells and fibroblasts (Fig. 4b), but no significant differences in immune cells (Fig. 4c). We further subcategorized immune cell subsets, combined with the results of multiplex immunofluorescence, focusing on T cell subsets. Based on canonical markers for T cells and noticed significant changes in the CD8T_01_GZMK_CXCR4 subset, while other subsets remained unchanged (Fig. 4d, e).

## Discussion

This is the initial phase II study to assess neoadjuvant IBC treatment in managing RCC with IVC-TT (Mayo level II–IV). After 12 weeks treatment of toripalimab plus axitinib, 44.0% (11/25) patients achieved a reduction in thrombus level, met the prespecified target of 30%. Twenty-four (96.0%) patients had reduction in TT length with a median 2.3 cm reduction, the ORR (complete response + PR) of TT was 44.0%. Moreover, 61.9% (13/21) patients experienced changes in surgical strategy compared with planned surgery, though some of them didn’t have a downgrading in TT. In total, the present study met preset endpoints and proved that the feasibility of applying IBC to downgrade TT level and reduce surgical coverage in cases with RCC and IVC-TT. Importantly, drug toxicity and morbidity were in line with former studies,^2,13^ and no patient experienced clinically related progression of TT.

Neoadjuvant therapy for RCC is often discussed in clinical practice. Due to the development trend of systemic agents, pre-surgical TKI in RCC with TT was more often reported in previous studies.^14^ Most of them were retrospective case report and series, a phase two trial firstly evaluated neoadjuvant axitinib in ccRCC with venous invasion. They have found that 35.0% (7/20) patients achieved a reduction in thrombus level: 37.5% (6/16) with IVC-TT and 25.0% (1/4) with renal vein TT.^9^ For locally advanced RCC and not limited to patients with TT, two phase II studies recently have evaluated the safety and feasibility of neoadjuvant nivolumab. Though radiographic changes in tumor diameter were minimal, most patients experienced tumor regression in nephrectomy specimen, which may due to the anticancer mechanism of immunotherapy by enhancing T-cell activation.^15,16^ Several case report and series have explored the safety and feasibility neoadjuvant IBCs in RCC with TT,^8,12^ however, the level of evidence was low. Our study met preset endpoints proving that toripalimab in combination with axitinib downstages IVC-TT in a significant proportion of patients leading to simplification in the procedure of surgery.

According to our results, the observed safety profile was generally consistent with the adverse event spectrum of toripalimab or axitinib monotherapy. The common TRAEs were fatigue, diarrhea, hypertension, proteinuria, in line with previous studies.^13^ Moreover, perhaps due to the shorter period of therapy, the rate of TRAEs was relatively lower than treatment in metastatic RCC.^13^ No patient delayed surgery because of any TRAE. These results further confirmed the safety of neoadjuvant toripalimab and axitinib.

Several factors were taken into account when determining the enrollment criteria for this study. Firstly, based on our previous studies about surgical techniques,^17,18^ we supposed that the downgrading of TT can significantly change the surgical approach in cases with former high-level TT. Moreover, presurgical targeted therapy was found to be more effective in downgrading high-level TT.^5^ As a result, only cases with Mayo level II–IV TT were included in the study. Secondly, while IBC achieved satisfactory outcomes in advanced non-clear cell RCC,^19^ the level of evidence supporting these findings is low. Therefore, only patients with ccRCC were considered for inclusion in the study. Thirdly, performing cytoreductive nephrectomy for metastatic RCC is a topic of controversy, particularly for poor-risk patients.^20^ In our study, all five metastatic patients belonged to either favorable or intermediate risk groups and achieved PR or stable disease following treatment. Four of them underwent radical surgery successfully, while one patient with level IV TT exhibited a poor response in TT and declined surgery due to the associated high risk.

It is meaningful to identify biomarkers of therapy effectiveness, which can guide strategy of neoadjuvant treatment. Pretreatment biopsy samples from tumor can directly present inherent characteristics of RCC patients. Since tumor thrombus can’t be punctured, we can only obtain biopsy samples from primary renal tumors. Previous studies have reported that TT and the matched primary tumors were molecularly alike.^21,22^ Based on Cell DIVE multiplexed imaging platform, our findings suggested that while NR patients show higher T-cell infiltration, these infiltrating cells may predominantly be immunosuppressive T cytotoxic cells expressing PD1. These PD1-expressing cells likely interact with PDL1-expressing tumor cells, ultimately leading to resistance to neoadjuvant therapy. Therefore, the preoperative assessment of T cytotoxic PD-1/+ cell levels in tumor puncture specimens may serve as a predictor of treatment response. In the study of neoadjuvant axitinib for RCC with TT, though statistical difference wasn’t observed, the authors have identified similar trend of T cells.^9^ Furthermore, single-cell RNA-seq (scRNA-seq) was performed in postoperative TT samples from NR and R patients. Based on canonical markers for T cells,^23^ we noticed significant changes in the CD8T_01_GZMK_CXCR4 subset. Previous studies have reported that CD8^+^ GZMK^+^ had the highest cytotoxic score and the lowest exhausted score, associating them with a favorable response to combined therapy.^23^ In our scRNA-seq samples, these cells were more ample in R patients compared to NR patients, suggesting a potential link to neoadjuvant therapy efficacy. In conclusion, our findings indicate that T cell subsets in both preoperative and postoperative samples may be associated with the effectiveness of neoadjuvant therapy in RCC-TT patients.

This study has several limitations that should be noted. The median follow-up time may not be sufficient to capture all disease progression. In particular, the relationship between tumor response and long-term survival needs further study. As only patients with ccRCC were enrolled in the trial, it should be caution about extending our findings to pre-treatment histological proof of non-clear cell RCC. Because of technical reasons, Cell DIVE multiplexed imaging and scRNA-seq were performed in samples from part of patients. Moreover, tumor thrombus can’t be punctured, we can only predict response of TT using preoperative renal tumor biopsy samples. It is crucial to expand the sample size and analyze the paired of preoperative and postoperative samples to further validate our findings. Lastly, ccRCC patients with high-risk recurrence can benefit from adjuvant pembrolizumab.^24^ However, the positive results were published after the initial time of our study. Preliminary results from other adjuvant ICI trials have been conflicting. Moreover, many factors may be taken into account when applying these results in clinical practice. Actually, there is no standard treatment for these RCC patients after neoadjuvant therapy. Therefore, no strict requirements were made for postoperative therapy in our study.

In conclusion, this phase two study demonstrated the combination of toripalimab plus axitinib shows promise in down-staging IVC-TT and reducing the need for extensive surgical procedures for patients with ccRCC and IVC-TT. The safety of this combination therapy also has been proved with the absence of TRAE leading to surgery delays. Additionally, T cell subsets in both preoperative and postoperative samples may be associated with the effectiveness of neoadjuvant therapy in RCC-TT patients.

## Materials and methods

### Trial oversight

This study was approved by the ethics committee of Chinese PLA General Hospital. The trial complied with the principles of Good Clinical Practice and the Declaration of Helsinki and was registered in the Chinese Clinical Trial Registry (No. ChiCTR2000030405).

### Patients

Eligible patients must be at least 18 years old, have previously untreated RCC, and have IVC-TT. For all cases, a preliminary diagnostic biopsy of renal lesions to affirm the histology of ccRCC was performed. Key inclusion criteria were level II–IV IVC-TT (cT3b/c), cN0/1, cM0/1, candidates for radical nephrectomy, and IVC thrombectomy, life expectancy is greater than 3 months. The included metastatic RCCs were oligometastatic patients, the number of metastases was limited and complete resection can be achieved by local treatment such as surgery. Additional inclusion criteria were Eastern Cooperative Oncology Group performance status 0 or 1; adequate bone marrow, kidney, and liver function as defined by: neutrophil ≥ 1.5 × 10*^9^/L, platelet ≥ 100 × 10*^9^/L, hemoglobin ≥ 90 g/L, serum creatinine ≤ 1.5 folds the upper limit of normal (ULN), alanine aminotransferase and aspartate aminotransferase ≤ 2.5 ULN. The primary exclusion criteria were previous anticancer systemic therapy including TKI or IBC, recent history of cardiac or vascular events, and M1 participants belonging to IMDC poor-risk group.

### Study design

The NEOTAX study, a phase 2 open-label single-arm trial, utilized a preoperative treatment regimen of toripalimab in combination with axitinib. Toripalimab was recommended to administer 240 mg every 3 weeks for 60 min by intravenous infusion, lasted up to four cycles. The dose of axitinib was 5 mg BID. Dose reductions were allowed. Response of tumor thrombus and overall tumor was assessed firstly before cycle three treatment, following treatment was given at the discretion of the investigators when tumor thrombus or overall tumor progressed, otherwise the treatment lasted till surgery (Fig. 1a).

### Endpoints and assessments

The primary endpoint was the downstaging rate of IVC-TT level after neoadjuvant toripalimab plus axitinib therapy. The stage of IVC-TT was assessed according to Mayo Clinic classification system (level 0: tumor thrombus confined to the renal vein; level 1: entering the IVC, less than 2 cm away from the opening of the renal vein; level 2: IVC extension > 2 cm from the opening of the renal vein, lower than the hepatic vein; level 3: tumor thrombus at or above hepatic vein level, but lower than diaphragm; level 4: the thrombus extends above the diaphragm).^2^ Secondary endpoints were the percentage and absolute reduction in IVC-TT length, overall tumor and TT response rate, percentage change in surgical approach, PFS (time from the onset of preoperative therapy to progression, recurrence, or death), overall survival (time from the onset of preoperative therapy to death), surgical morbidity by Clavien–Dindo classification, and safety. The exploratory endpoint was biomarker analysis of the efficacy of toripalimab plus axitinib.

Patients were evaluated clinically and for safety on schedule according to protocol (supplementary data). All the adverse events were graded based on National Cancer Institute-Common Terminology Criteria for Adverse Events (version 5.0). Radiological evaluation was performed before initial therapy, before cycle 3 of toripalimab and before operation by MRI scans.

### Surgery and change of approach

The detailed surgical procedures were described in our previous publications.^18,25,26^ Percentage change of surgical approaches was calculated through comparing planned surgical approaches reported by surgeons with those performed according to three parts: 1. Shift from “open surgery” to “minimally invasive surgery”; 2. Shift from a more invasive surgical approach to less invasive approach, including avoid cardiopulmonary bypass, less portal blood vessel occlusion, less liver mobilization, less extensive surgical incision; 3. Avoiding intraoperative patient-position change.

### Cell DIVE multiplexed imaging

The Cell DIVE imaging process refer to published articles.^27,28^ The supplementary materials provide a detailed protocol. Due to tissue detachment, 13 samples were finally included. Follow-up analysis was conducted using HALO software.

### Single-cell RNA-seq and analysis

We performed scRNA-seq on seven surgical samples of tumor thrombus. Using SeekOne® MM Single Cell 3’technology for scRNA-seq.^29,30^ Detailed procedures were provided in the supplementary material.

### Statistical analysis

Simon’s two-stage minimax design^31^ was utilized to differentiate between a cohort reduction in the Mayo level of ≤10% and ≥30%, requiring a sample size of 25 evaluable patients (90% power, one-sided alpha of 0.1). To determine the clinical trial achieving a success, a reduction in the Mayo level should be observed in at least 4 of the 25 evaluable patients following 12 weeks of treatment. In the first stage, 16 patients needed to be enrolled. If the reduction in Mayo level was observed in no more than one patient, recruitment to the present trial would close. If ≥2 patients were observed a reduction in the TT level after 12 weeks treatment, the additional nine patients would be enrolled.

Patients who received one or more cycle of toripalimab with any dose of axitinib and underwent on-study disease evaluation before surgery were included for efficacy analysis. Patients who received one or more dose of either toripalimab or axitinib were included for safety analysis. According to RECIST evaluation, patients with complete and PR in TT were defined as R, the rest patients were defined as NR. The number of cell types in puncture formalin-fixed, paraffin-embedded renal tumor samples and postoperative TT samples were compared between R and NR to explore biomarkers associated with therapy efficacy. For the comparison of Mayo level of IVC-TT before and after neoadjuvant therapy, the marginal homogeneity test was adopted. PFS was estimated using Kaplan–Meier curve. The statistical analyses were conducted using R version 4.2.1.

## Supplementary information

Supplementary materials

Study protocol

## Data Availability

The raw data originating from this research have been archived in the Genome Sequence Archive, accessible under the identifier HRA006643. Given the nature of these data involving human genetic resources, interested parties may acquire the raw data within a six-month timeframe by adhering to the specified guidelines for non-commercial use at the Genome Sequence Archive, which can be found at https://ngdc.cncb.ac.cn/search/?dbId=hra&q=HRA006643. Moreover, to ensure transparency and reproducibility, all the computational scripts and codes can be found on Github at https://github.com/zhangqi234/PLAGHNeo.
