# Supplementary material for: Neoadjuvant toripalimab plus axitinib for clear cell renal cell carcinoma with inferior vena cava tumor thrombus: NEOTAX, a phase 2 study
Source: Signal Transduct Target Ther. 2024 Oct 4;9:264. doi: 10.1038/s41392-024-01990-2 (PMC11450193; doi:10.1038/s41392-024-01990-2)
Supplement: Supplementary file 1 — Supplementary materials [file 41392_2024_1990_MOESM1_ESM.docx]

Supplementary Materials for

Neoadjuvant toripalimab plus axitinib for clear cell renal cell carcinoma with inferior vena cava tumor thrombus: NEOTAX, a phase 2 study

Liangyou Gu, Cheng Peng, Qiyang Liang, Qingbo Huang, Deqiang Lv, Houming Zhao, Qi Zhang, Yu Zhang, Peng Zhang, Shichao Li, Junnan Xu, Luyao Chen, Yongpeng Xie, Jinhang Li, Gang Guo, Xu Zhang, Baojun Wang, Xin Ma

Correspondence to: [xzhang301@163.com](mailto:xzhang301@163.com)

**This PDF file includes:**

Materials and Methods

Figure. S1

Table. S1

Materials and Methods

Cell DIVE multiplexed imaging

Tissue sections were baked for overnight at 60℃, deparaffinized, hydrated, processed through a two-step antigen retrieval process (step 1: citrate-based pH 6.0, Vector, cat. #H-3300; step 2: EDTA-based pH 8.8-9.0, Sigma, cat. #T6066-100G/Bio-Rad, cat. # 161-0729/Sigma, cat. #P9416) using a previously published protocol, and blocked one hour using 3% BSA (sigma, cat. #B2064). The tissue was then stained with DAPI for 15 minutes (Thermo Scientific, cat. #D3571) and washed three rounds for five minutes with 1×PBS. A whole slide image was acquired for field-of-view selection. Tissue sections then underwent 9 cycles of background imaging, staining, imaging, and signal inactivation. Marker Panel: CD57, CD11b, CD68, PanCK, SMA, CD33, HLA-DR, CD14, CD163, CD45RO, CD68, PD-1, PD-L1, CD20, FoxP3, CD8, CD3, CD4, CD31.

Images were acquired using the Cell DIVE (Leica Microsystems). Image App software was used for image acquisition and registration (using DAPI). An acquired background image following each cycle of dye inactivation was used to subtract autofluorescence from the subsequent stain round resulting in autofluorescence removed images. Due to tissue detachment, 13 samples were finally included. Follow-up analysis was conducted using HALO software.

Single cell RNA-seq and analysis

We performed scRNA-seq on seven surgical samples of tumor thrombus. After harvest, tissues were washed in ice-cold RPMI-1640 and dissociated using Tissue Dissociation Reagent A (Seekone K01301-30) from SeekGene as instructions. DNase Ⅰ (Sigma 9003-98-9) treatment was optional according to the viscosity of the homogenate. Cell count and viability was estimated using fluorescence Cell Analyzer (Countstar® Rigel S2) with AO/PI reagent after removal erythrocytes (Solarbio R1010) and then debris and dead cells removal was decided to be performed or not (Miltenyi 130-109-398/130-090-101). Finally fresh cells were washed twice in the RPMI1640 and then resuspended at 1×106 cells per ml in 1×PBS and 0.04% bovine serum albumin.

Single-cell RNA-Seq libraries were prepared using SeekOne® MM Single Cell 3’ library preparation kit (SeekGene Catalog No. K00104). Briefly, appropriate number of cells were loaded into the flow channel of SeekOne® MM chip which had 170,000 microwells and allowed to settle in microwells by gravity. After removing the unsettled cells, sufficient Cell Barcoded Magnetic Beads (CBBs) were pipetted into flow channel and also allowed to settle in microwells with the help of a magnetic field. Next excess CBBs were rinsed out and cells in MM chip were lysed to release RNA which was captured by the CBB in the same microwell. Then all CBBs were collected and reverse transcription were performed at 37℃ for 30 minutes to label cDNA with cell barcode on the beads. Further Exonuclease I treatment were performed to remove unused primer on CBBs. Subsequently, barcoded cDNA on the CBBs was hybridized with random primer which had reads 2 SeqPrimer sequence on the 5’ end and could extend to form the second strand DNA with cell barcode on the 3’ end. The resulting second strand DNA were denatured off the CBBs, purified and amplified in PCR reaction. The amplified cDNA product was then cleaned to remove unwanted fragment and added to full length sequencing adapter and sample index by indexed PCR. The indexed sequencing libraries were cleanup with SPRI beads, quantified by quantitative PCR (KAPA Biosystems KK4824) and then sequenced on illumina NovaSeq 6000 with PE150 read length.

The raw data of scRNA-seq in this study were deposited in Genome Sequence Archive with accession ID HRA006643. Since these data are related to human genetic resources, raw data can be obtained within half year by requesting and following the guidelines for Genome Sequence Archive for noncommercial use at https://ngdc.cncb.ac.cn/search/?dbId=hra&q= HRA006643. All raw scripts and codes used to analyze data and generate figures are available at Github (https://github.com/zhangqi234/PLAGHNeo). The data that support the main findings of this study and additional information are also available from the author (Dr. Qi Zhang) upon request.


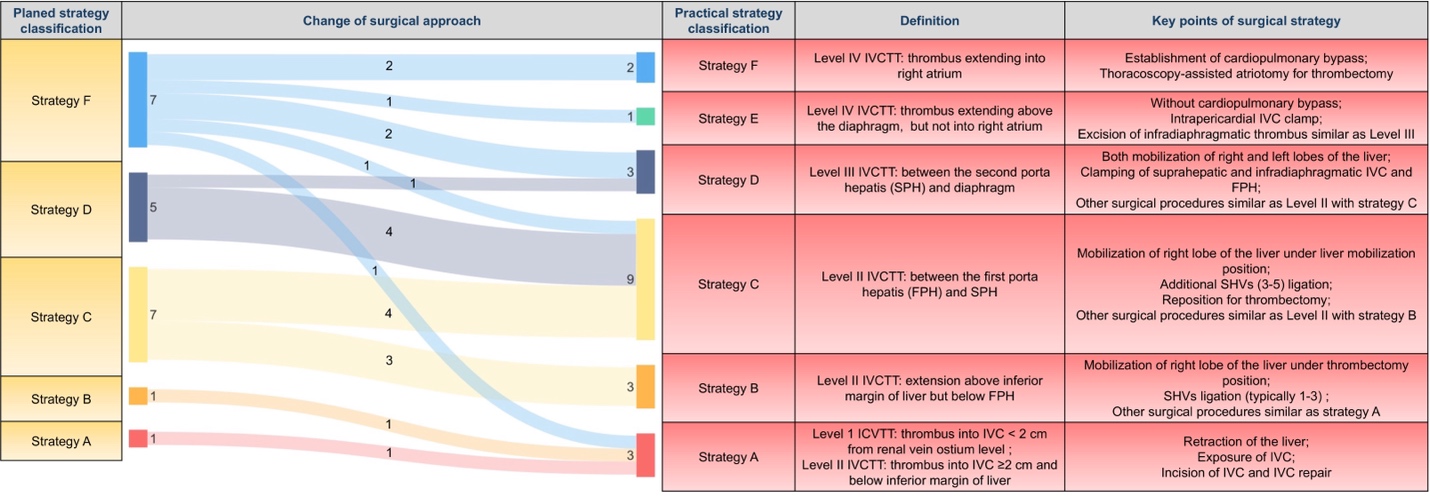


Figure. S1.

Surgical strategy classification and sankey diagram of changes in surgical approach. We used sankey diagram to demonstrate changes in surgical approach. Based on Mayo classification and specific anatomical landmarks, we divided robot-assisted IVC tumor thrombectomy into 6 different surgical strategies (strategy A-F). With the upgrading of surgical strategy classification, the surgical complexity and steps increased. The definition of strategy classification and key points of surgical strategy were described. IVC, inferior vena cava; IVCTT, IVC tumor thrombus; SHV, short hepatic veins; FPH, first porta hepatis; SPH, second porta hepatis.

Table S1. Cell markers of cell dive.

| **Cell Type** | **Marker** |
| --- | --- |
| B cell | CD20+ |
| B cell PD-L1/- | CD20+PDL1- |
| B cell PD-L1/+ | CD20+PDL1+ |
| ECs | CD31+ |
| Fibroblast | SMA+ |
| M1 Macrophage | CD163-CD68+ |
| M1 Macrophage PD-L1/- | PDL1-CD163-CD68+ |
| M1 Macrophage PD-L1/+ | PDL1+CD163-CD68+ |
| M2 Macrophage | CD163+CD68+ |
| M2 Macrophage PD-L1/- | PDL1-CD163+CD68+ |
| M2 Macrophage PD-L1/+ | PDL1+CD163+CD68+ |
| Macrophage | CD68+ |
| MDSC | CD11b+CD33+HLA-DR- |
| Monocyte | CD11b+CD14+ |
| Myeloid cell | CD11b+ |
| Natural Killer cell | CD3-CD57+ |
| Neutrophil/G-MDSC | CD11b+HLA-DR-CD16+ |
| T cell | CD3+ |
| T cytotoxic | CD3+CD4-CD8+CD45RO- |
| T cytotoxic memory | CD3+CD4-CD8+CD45RO+ |
| T cytotoxic memory PD-1/- | CD3+PD1-CD4-CD8+CD45RO+ |
| T cytotoxic memory PD-1/+ | CD3+PD1+CD4-CD8+CD45RO+ |
| T cytotoxic PD-1/- | CD3+PD1-CD4-CD8+CD45RO- |
| T cytotoxic PD-1/+ | CD3+PD1+CD4-CD8+CD45RO- |
| T helper | CD3+CD4+CD8-FOXP3-CD45RO- |
| T helper memory | CD3+CD4+CD8-FOXP3-CD45RO+ |
| T helper memory PD-1/- | CD3+PD1-CD4+CD8-FOXP3-CD45RO+ |
| T helper memory PD-1/+ | CD3+PD1+CD4+CD8-FOXP3-CD45RO+ |
| T helper PD-1/- | CD3+PD1-CD4+CD8-FOXP3-CD45RO- |
| T helper PD-1/+ | CD3+PD1+CD4+CD8-FOXP3-CD45RO- |
| T regulatory | CD3+CD4+CD8-FOXP3+CD45RO- |
| T regulatory PD-1/- | CD3+PD1-CD4+CD8-FOXP3+CD45RO- |
| T regulatory PD-1/+ | CD3+PD1+CD4+CD8-FOXP3+CD45RO- |
| Tumor cell | PANCK+ |
| Tumor cell PD-L1/- | PDL1-PANCK+ |
| Tumor cell PD-L1/+ | PDL1+PANCK+ |
