# Supplementary material for: Neoadjuvant toripalimab plus axitinib for clear cell renal cell carcinoma with inferior vena cava tumor thrombus: NEOTAX, a phase 2 study
Source: Signal Transduct Target Ther. 2024 Oct 4;9:264. doi: 10.1038/s41392-024-01990-2 (PMC11450193; doi:10.1038/s41392-024-01990-2)
Supplement: Supplementary file 2 — Study protocol [file 41392_2024_1990_MOESM2_ESM.pdf]

A Phase II Trial of Neoadjuvant Toripalimab plus Axitinib for Clear Cell Renal Cell Carcinoma with Inferior Vena Cava Tumor Thrombus

Study Protocol

Investigating Institution: Chinese PLA General Hospital

Principal Investigator: Professor Xu Zhang

Version/Date: Version 1.0/Nov. 22, 2019

Confidentiality Statement: All information contained in this protocol is proprietary to the PLA General Hospital and will only be provided to the investigators, co-investigators, ethics committees, regulatory authorities and other relevant authorities for review. Without the written approval of the PLA General Hospital, no third party not involved in this study should be informed of any information except when signing an informed consent form with a subject who may participate in this study and giving them the necessary explanation.

Protocol Synopsis

**Study Title:**	A Phase II Trial of Neoadjuvant Toripalimab plus Axitinib for Clear Cell Renal Cell Carcinoma with Inferior Vena Cava Tumor Thrombus
**Study Phase:**	Phase II
**Enrolled Population:**	Renal cell carcinoma with Mayo II-IV inferior vena cava tumor thrombus (T3b-T3c), with or without lymph node or distant metastasis (N0/1, M0/1), male or female, aged ≥ 18 years; candidate patients for surgical treatment (radical nephrectomy combined with inferior vena cava thrombectomy) and life expectancy is greater than 3 months; biopsy before treatment confirmed clear cell renal cell carcinoma.
**Primary Study Endpoint**	Down-staging rate of tumor thrombus: evaluated according to Mayo Clinic and 301 classification system.
**Secondary Study Endpoints:**	Percentage change in tumor thrombus height and percentage change in tumor thrombus volume;<br>Response rate evaluated by RECIST version 1.1;<br>PFS (time from the onset of preoperative therapy to progression, recurrence, or death), OS (time from the onset of preoperative therapy to death);<br>Percentage change in surgical approach;<br>Incidence of surgical complications assessed based on Clavien-Dindo complication grading system;<br>Incidence and severity of AEs and SAEs based on National Cancer Institute-Common Terminology Criteria for Adverse Events (NCI-CTCAE) 5.0;<br>Biomarker analyses of the effect of toripalimab in combination with axitinib.
**Study Design:**	The study was a phase II, open-label, single-arm prospective cohort study in which patients who met the inclusion and did not meet exclusion criteria received targeted combined immunotherapy for

	12 weeks, axitinib 5 mg bid combined with toripalimab 240 mg q3w. After 6 weeks of treatment, the effect of drug treatment was assessed for the first time. If tumor thrombus progressed, hospitalization was required immediately for surgical treatment, otherwise the treatment lasted for 12 weeks.

1. Background

1.1 Background of Preoperative Drug Therapy for Renal Cell Carcinoma with Venous Tumor Thrombus

Renal cell carcinoma (RCC) is a common malignant tumor of the genitourinary system, accounting for approximately 2%-3% of adult malignant tumors. Renal cell carcinoma is characterized by easy invasion into the venous system to form tumor thrombus, and it is reported in the literature that about 4%-10% of RCC patients have inferior vena cava tumor thrombus[1]. Radical nephrectomy combined with caval thrombectomy is the preferred treatment for patients with renal cell carcinoma and vena cava tumor thrombus, and is one of the operations with the highest-risk in urology, often facing complications such as massive hemorrhage and thrombosis detachment. The previous studies showed that the operative mortality of patients with tumor thrombus was 5%-10%, with the overall complication rate of 38%, the higher the level of tumor thrombus, and the higher the incidence of perioperative complications.

In recent years, many scholars have explored the efficacy and safety of preoperative drug therapy for locally advanced renal cell carcinoma. In the era of targeted therapy, a number of retrospective studies have confirmed that preoperative targeted therapy has a certain tumor-shrink effect, but the overall degradation rate of tumor thrombus is not high. Advanced kidney cancer has gradually entered the era of immunotherapy since the PD-1 inhibitor nivolumab (NIVO) was approved for second-line treatment of advanced renal cancer[7] in 2015. Several phase III clinical studies have also confirmed the significant efficacy of PD-1 monoclonal antibody combined with targeted drugs in the first-line treatment of advanced renal cell carcinoma, and the objective response rate is superior to targeted therapy alone.

At present, there have been no clinical studies report on preoperative immunotherapy or immune combination therapy in this field of renal cell carcinoma. Individual case reports describe that preoperative neoadjuvant immunotherapy can achieve significant tumor-shrinking effects and reduce the difficulty and risk of renal cell carcinoma with inferior vena cava tumor thrombus surgery. In order to further confirm the tumor shrinkage effect of targeted combination immunotherapy in renal cell carcinoma and the impact on the overall survival of patients, we have initiated an open-label, single-arm clinical study of preoperative axitinib combined with toripalimab in the treatment of renal cell carcinoma with inferior vena cava tumor

thrombus.

1.2 Introduction of Study Drugs

1.2.1 Axitinib

Axitinib, a multi-targeted receptor tyrosine kinase inhibitor that selectively inhibits VEGFR 1-3, has been approved for the treatment of advanced renal cell carcinoma after failure of first-line systemic therapy (actual approved indications vary by country and region).

Two randomized Phase 3 clinical studies have assessed the efficacy of axitinib monotherapy in advanced renal cell carcinoma. Patients with advanced renal cancer who failed systemic therapy were treated with either axitinib or sorafenib. The primary endpoint, median progression-free survival (PFS), showed that axitinib was statistically significantly superior to sorafenib, with PFS of 6.7 months and 4.7 months respectively (HR: 0.67; 95% CI: 0.54-0.81; P<0.0001). There was no difference in median overall survival (OS) between the two treatment groups (axitinib was 20.1 months and sorafenib was 19.2 months; HR: 0.969; 95% CI: 0.800-1.174; one-sided P=0.3744). Axitinib has been approved by the FDA and EMA for the treatment of advanced renal cell carcinoma following prior treatment failure [8,9].

In the Phase III study of axitinib versus sorafenib in untreated advanced clear cell renal cell carcinoma, 2:1 randomization (axitinib: N=192, sorafenib: N=96) was performed. There was no significant difference in median PFS between the axitinib and sorafenib groups, which were 10.1 months (95% CI 7.2-12.1) and 6.5 months (95% CI 4.7-8.3), respectively, with a stratified HR of 0.77 (95% CI, 0.56-1.05). The independent review committee assessed that the ORR in the axitinib arm (32%) was statistically significant higher compared with the ORR in the sorafenib arm (15%), with a HR of 2.21 (95% CI, 1.31-3.75, one-sided p-value of the stratum = 0.0006)[10] . Based on these data, the NCCN guidelines recommended axitinib as a first-line treatment option for advanced renal cell carcinoma[11] .

The most common adverse events (≥20%) observed with axitinib include diarrhea, hypertension, weight loss, fatigue, decreased appetite, hand-foot syndrome, dysphonia, asthenia, hypothyroidism, and nausea[10] . Overall, the adverse events reported with axitinib in clinical studies are considered manageable and generally reversible. Further details on clinical pharmacology and safety are provided in the axitinib package insert.

1.2.2 Toripalimab

Toripalimab injection, hereafter referred to as JS001, targets human PD-1 and is a neutralizing blocking antibody. It binds to PD-1 with high affinity and selectively blocks the binding of PD-1 to its ligands PD-L1 and PD-L2, thereby activating T lymphocytes and improving lymphocyte proliferation as well as cytokine secretion, especially IFN-γ.

Preclinical pharmacodynamic studies have confirmed that JS001 significantly stimulates the proliferation of CD4 + and CD8 + T cells and promotes the activation of human effector/memory T cells in animal models of graft-versus-host disease (GVHD) induced by adoptive transfer of human PBMCs (peripheral blood mononuclear cells). At the same time, studies in the animal model of transplanted human melanoma 624 MEL have demonstrated that JS001 combined with killer lymphocytes (CTLs) can eliminate the immunosuppressive effect and promote the killing effect of CTLs on tumor cells, achieving the expected good therapeutic effect.

Compared with nivolumab and pembrolizumab, the differences between JS001 and the antigen-binding sites' CDR sequences and structures are mainly reflected in the differences in six CDR sequences, which determine the different physicochemical and biological characteristics of the three. Based on SPR or ELISA affinity testing results, JS001 has higher affinity than pembrolizumab and nivolumab, as shown in Table 1.

Table 1 Comparison of structure and physicochemical properties between JS001 and drugs with the same target

Sample	Antibody subtype		Affinity (SPR)	Binding EC50(Elisa)	Source
	Heavy chain	Light chain			
JS001	IgG4	Kappa	0.92nM	64pM	Humanized
Pembrolizumab	IgG4	Kappa	Not reported	70pM	Humanized
Nivolumab	IgG4	Kappa	2.64nM	Not reported	Fully humanized

JS001 drug product is developed as a solution for injection in a strength of 240 mg/6 ml per vial. On December 23, 2015, CFDA Drug Clinical Trial Approval Letter No.: 2015L05752 was obtained.

a) Pharmacodynamics

In vitro pharmacodynamic studies have shown that antigen memory response JS001 significantly stimulated T cell proliferation and promoted IFN-γ release. The results of in vivo experiments in NSG mice showed that JS001 could effectively increase the proliferation of human CD4 + and CD8 + T cells in vivo, while better promoting the activation of human effector/memory T cells, and its stimulating effect was significantly stronger than that of nivolumab, a similar target drug marketed abroad. JS001 can eliminate the immune suppression of T cells by tumors and promote the killing effect of cytotoxic T lymphocytes (CTLs) on tumor cells in vivo. In addition, JS001 did not cause antibody-dependent cell-mediated cytotoxicity or cytokine storms.

Receptor occupancy assay in cynomolgus monkeys showed that JS001 bound to PD-1 on the cell surface in a dose-dependent manner, and the effective dose started at an antibody serum concentration of 0.3 μg/mL and reached saturation at 3 μg/mL, exhibiting optimal biological effect. These preclinical data provide a basis for dose extrapolation to support further clinical studies with JS001.

b) Pharmacokinetics/Pharmacodynamics and Immunogenicity

Following a single intravenous infusion of different doses (1, 10, 75 mg/kg) of the test drug JS001 in cynomolgus monkeys, serum drug exposure levels basically increased linearly with dose over the dose range of 1 to 10 mg/kg. C_{max} was 27.70 ± 12.29 μg/mL, 216.11 ± 34.52 μg/mL, and 1891.72 ± 270.16 μg/mL in each dose group, respectively. The vivo t1/2 was ranged from 134 to 194 hours, which was similar to the reported pharmacokinetic (PK) data for nivolumab and pembrolizumab, and no significant immunogenicity was found.

c) Toxicology

JS001 had no specific binding to murine PD-1, and cynomolgus monkeys were selected as the main test animals in toxicology studies by referring to the Guidelines for Non-clinical Safety Evaluation of Therapeutic Biological Products. National (Shanghai) Center for Safety Evaluation and Research of New Drugs was entrusted to conduct systematic preclinical safety toxicological study and evaluation in strict accordance with the requirements of Good Laboratory Practice for Non-Clinical Laboratory Studies. General pharmacology, acute toxicity, long-term toxicity testing, hemolysis and local irritation and other toxicity studies were performed.

In safety pharmacology evaluation studies of recombinant humanized anti-PD-1 antibodies, intravenous administration of JS001 to cynomolgus monkeys at doses of 10,

30, and 100 mg/kg had no significant effect on the cardiovascular system and respiratory rate of animals. Meanwhile, JS001 had no effect on motor coordination and behavioral activity in rats.

The acute toxicity test showed that a single intravenous injection of JS001 in cynomolgus monkeys showed no significant acute toxic effects, and the no-observed-adverse-effect level (NOAEL) was 406 mg/kg.

In the subacute toxicity test of repeated intravenous injection of JS001 once a week for four consecutive weeks, no significant toxic side effects were observed in JS001. No treatment-related adverse reactions were observed in any treated animals. No regular changes of toxicological significance were observed in body weight, body temperature, electrocardiogram (ECG) parameters, coagulation function, ophthalmic examination, urine routine distribution, serum cytokines and various blood biochemical indicators at each test time point in each dose group. JS001 exhibited linear kinetics in cynomolgus monkeys with accumulation in the body after repeated dosing.

During the long-term toxicity test of JS001 at doses of 10, 30 and 100 mg/kg in cynomolgus monkeys intravenously injected for 26 consecutive weeks, no JS001-related abnormal changes in body weight, food consumption, clinical observation, body temperature, ECG, ophthalmology, blood routine and coagulation function, serum biochemistry, immune function, urine and fecal examination, gross pathology, organ weight, and histopathology showed no significant toxic reactions. Therefore, the NOAEL for JS001 was 100 mg/kg.

Detailed information about JS001 preclinical studies can be found in the JS001 Investigator 's Brochure.

d) Previous Clinical Experience in Toripalimab Injection (JS001)

As of 16 December 2019, 11 Phase 1 clinical studies, 5 Phase 2 clinical studies, and 10 Phase 3 clinical studies have been conducted in China; 1 Phase 1 clinical study has been conducted in the United States as shown in Table 2.

Table 2 Performed Clinical Trials of Toripalimab

Tumor type/ Study Code	Investigations Stage	Study Population	Planned/Actual Enrolled	Investigations Status
Advanced solid tumors				

CT1	I	Advanced solid tumors	36/36	Enrollment completed
CT2	I	Advanced solid tumors	12/25	Enrollment completed
CT3	I	Advanced solid tumors	36/33	Enrollment completed
Lymphoma				
CT6	I	Relapsed refractory malignant lymphoma	12/13	Enrollment completed
Neuroendocrine tumor				
CT14	I	Advanced Neuroendocrine Tumors Following Failure of Standard Therapy	40/36	Enrollment completed
Multiple cancer species multiple cohort				
CT5	Ib/II	Advanced gastric adenocarcinoma, esophageal squamous cell carcinoma, nasopharyngeal carcinoma, head and neck squamous cell carcinoma	Nasopharyngeal carcinoma combined 12/12	Enrollment completed
			Esophageal squamous cell carcinoma alone 58/60	
			Esophageal squamous carcinoma combined 12/12	
			Gastric cancer alone 58/58	
			Gastric cancer combined 36/33	
			Squamous cell carcinoma of head and neck 30/34	
			Head and neck squamous cell carcinoma combined 12/3	
Triple negative breast cancer				
CT9	I	Advanced triple negative breast cancer	24/20	Enrollment completed

CT10	I	Advanced triple negative breast cancer (combined radiotherapy)	30/6	In Enrollment
CT26	III	1L/2L TNBC	660/74	In Enrollment
Melanoma				
CT7-2	I	Investigate pharmacokinetic similarity before and after process changes in advanced melanoma patients	24/26	Enrollment completed
CT13	I	Advanced renal cell carcinoma and melanoma (in combination with axitinib)	24/33	Enrollment completed
CT4	II	Local progression or metastatic melanoma after failure of standard therapy	120/128	Enrollment completed
CT8	II	Adjuvant therapy for completely resected mucosal melanoma	148/145	In Enrollment
CT17	III	First-line treatment of unresectable or metastatic melanoma	230/123	In Enrollment
Urothelial carcinoma				
CT12	II	Local progression or metastatic urothelial carcinoma after failure of standard therapy	150/151	Enrollment completed
Nasopharyngeal carcinoma				
CT15	III	1L NPC	280/291	Enrollment completed

Esophageal cancer				
CT21	III	Advanced or metastatic esophageal squamous cell carcinoma without prior systemic chemotherapy	500/133	In Enrollment
Liver cancer				
CT16	III	Adjuvant therapy after radical resection of liver cancer	402/74	In Enrollment
CT20	Ib	Neoadjuvant therapy for hepatocellular carcinoma	20/1	In Enrollment
CT37	III	First-line advanced hepatocellular carcinoma	-	Not enrolled yet
**NSCLC**				
CT7-1	I	Investigate pharmacokinetic similarity before and after process changes in advanced NSCLC patients	30/41	Enrollment completed
CT18	II	EGFR mutation-positive, EGFR-TKI-failed advanced NSCLC	40/40	Enrollment completed
CT19	III	1L NSCLC (squamous cell carcinoma + adenocarcinoma)	450/146	In Enrollment
CT25	III	1L NSCLC failed EGFR-TKI	350/39	In Enrollment
CT29	III	Perioperative systemic therapy for stage IIIA NSCLC	406/-	Not enrolled yet
**SCLC**				
CT28	III	1L SCLC	420/14	In Enrollment

Clinical Trials Conducted in the US				
TAB001-01	I	Advanced solid tumors	Part A 18ampB 240 /109	In Enrollment

1.2.3 The rationale for anti-PD-1 antibody combined with anti-angiogenic targeted therapy in advanced renal cell carcinoma

T cell-mediated anti-tumor immune processes include tumor antigen release and presentation, effector T cell activation and proliferation, migration of activated effector T cells into the tumor microenvironment, binding of activated effector T cells and killing tumor cells. In the tumor microenvironment, the binding of PD-L1 expressed by tumor cells to PD-1 on T cells inhibits T cell immunocompetence, thereby achieving immune escape. Anti-PD-1/L1 antibody can kill tumor cells by blocking the binding of PD-1 to PD-L1 and restoring the immune activity of T cells. Previous studies have found that PD-L1 expression in renal cell carcinoma is associated with poor prognosis. In addition, the relatively high mutation load in renal cell carcinoma suggests the presence of more tumor antigens that can be recognized by the immune system, which supports the application of immunotherapy in advanced renal cell carcinoma.

Mutations in the VHL gene are the most common genetic abnormalities in renal cell carcinoma, and inhibition or inactivation of the VHL gene leads to abnormal activation of VEGF-related signaling pathways, and abnormal VEGF can inhibit anti-tumor immune effects through multiple mechanisms. Firstly, VEGF directly inhibits dendritic cell maturation, thereby interfering with tumor antigen presentation and T cell activation. Secondly, abnormal angiogenesis caused by VEGF affects the migration and infiltration of T cells into the tumor site by altering the normal structure and function of blood vessels in the tumor microenvironment. Finally, in the tumor microenvironment, VEGF can directly inhibit the function of effector T cells, and recruit regulatory T cells or bone marrow-derived suppressor cells (MDSCs) to indirectly down-regulate the function of effector T cells.

In summary, based on the mechanisms of action of anti-tumor immunotherapy and anti-angiogenic targeted therapy targeted therapy, combining anti-PD-1 with anti-angiogenic targeted therapy targeted therapy can exert a synergistic anti-tumor effect in renal cancer.

2. Study Objectives

To investigate the clinical efficacy and safety of preoperative axitinib combined with toripalimab in patients with renal cell carcinoma and inferior vena cava tumor thrombus, to identify immunological and genomic predictors associated with treatment response, and preliminarily to explore the potential molecular mechanisms associated with drug resistance.

(1) Primary study objective: to evaluate the degradate rate of inferior vena cava tumor thrombus after neoadjuvant toripalimab plus axitinib therapy according to Mayo Clinic and 301 classification system, and to analyze the clinical efficacy of preoperative targeted combined immunotherapy.

(2) Secondary study objectives:

① To evaluate the efficacy of preoperative targeted combined immunotherapy on inferior vena cava tumor thrombus shrinkage based on imaging assessment by calculating the percentage and absolute change in tumor thrombus height, percentage change in tumor thrombus volume;

② To evaluate the efficacy of preoperative targeted combined immunotherapy on overall tumor control and its impact on the prognosis of patients with renal cell carcinoma and inferior vena cava tumor thrombus by ORR, DCR, PFS and OS based on RECIST v1.1;

③ To further evaluate whether preoperative combined therapy can reduce the difficulty and complexity of tumor thrombus surgery by the percentage of change in surgical decision-making;

④ To evaluate the effect of preoperative targeted combined immunotherapy on surgical safety through the incidence of surgical complications (Clavien-Dindo complication grading system);

⑤ To report the incidence and severity of AEs and SAEs (NCI-CTCAE 5.0) and evaluate the drug safety of preoperative targeted combined immunotherapy for patients with tumor thrombus;

⑥ To analyze biomarkers of the effect for toripalimab in combination with axitinib.

3. Study Design

3.1 Protocol Introduction

This trial was conducted by outpatient recruitment. The initial diagnosis of renal cell carcinoma with inferior vena cava tumor thrombus was based on imaging examination and laboratory test results. Histological evidence was obtained by percutaneous renal biopsy. After signing the informed consent form, patients who met the inclusion criteria and did not meet the exclusion criteria are treated with preoperative targeted combined immunotherapy. The treatment regimen for patients is as follows: axitinib starting dose was 5 mg, orally, bid, with an interval of 12 hours between doses; toripalimab 240 mg, intravenously, Q3W, lasts up to 4 cycles. Drug dose adjustment was performed by the research doctor according to the type and grade of adverse reactions in patients.

The patient's medical history, physical examination, drug regimen, CT/MRI examination, and laboratory tests are recorded or performed before the screening period. Laboratory tests including blood and urine routine, blood biochemistry, coagulation blood routine, and thyroid function were repeated at weeks 3, 6, 9, and 12 of treatment, and the dose, timing, and occurrence of adverse reactions of treatment are followed up by outpatient visits or telephone call before each immunotherapy dose cycle.

Patients will undergo chest and abdominal CT/MRI in the 6th and 12th weeks, and preoperatively. These will be independently assessed by an experienced radiologist (single-blind) for changes in the maximum diameter or height of the primary tumor and tumor thrombus and efficacy assessment according to RECIST1.1. According to imaging assessment after 6 weeks of treatment, if there is significant progression in tumor thrombus height or grade, hospitalization surgery is required immediately, otherwise preoperative assessment will be performed from treatment to 12 weeks.

After patients have been treated for 12 weeks and successfully completed preoperative targeted combined immunotherapy, the research doctor will decide whether to perform surgical treatment and the choice of surgical strategy through preoperative detailed evaluation. For patients receiving surgical treatment, the targeted drug was stopped for 36-48h before surgery. Postoperative specimens of the primary tumor and tumor thrombus were routinely sent for pathological examination to obtain a more exact pathological diagnosis, and the pathological response rate was analyzed by pathologists. For patients with tolerable adverse reactions in neoadjuvant therapy, maintenance therapy with toripalimab may be continued for 1 year within 3 months after surgery until disease progression or discontinuation due to serious adverse events,

as assessed by the treating physician. Patients were followed for at least 1 year after surgery and were seen in the clinic every 3 months for 1 year and by telephone calls or visits every 3 months for a total follow-up frequency of approximately 4 visits per year from the date of surgery.

3.2 Study Subjects

Patients with renal cell carcinoma and inferior vena cava tumor thrombus who met the inclusion criteria and did not meet the exclusion criteria (disease diagnosis was based on the imaging and laboratory parameters recommended by the guidelines and confirmed by needle biopsy).

3.3 Inclusion Criteria

1) Male or female, aged ≥ 18 years;
2) Renal cell carcinoma with level II-IV IVC tumor thrombus (T3b-T3c) with or without lymph node and distant metastasis (N0/1, M0/1);
3) Patients were candidates for surgical treatment (radical nephrectomy combined with inferior vena cava thrombectomy) and had a life expectancy of more than 3 months;
4) Clear cell renal cell carcinoma confirmed by renal biopsy before medication;
5) No previous systemic anti-cancer therapy including targeted therapy or immunotherapy;
6) ECOG status of 0 or 1;
7) Laboratory tests met medication criteria (specific criteria are listed in the case report form);
8) No surgical contraindications;
9) Patients have signed an informed consent form and agree to receive treatment.
10) Females of childbearing potential (defined as those who have not been sterilized and have been menopausal for less than 1 year) must provide a negative urine or serum pregnancy test within 4 weeks of registration and be retested on the day of treatment initiation. Fertile couples must terminate their fertility program during pregnancy;
11) Males (who have not undergone menopausal surgery) or females of childbearing potential agree to take necessary contraceptive measures during treatment (time frame defined as starting enrollment through 1 month after discontinuation);
12) Treatment with low molecular weight heparin, low dose anticoagulation to

maintain central venous patency and prevent deep vein thrombosis is allowed.

3.4 Exclusion Criteria

1) Unable to receive preoperative biopsy or adhere to medical treatment;
2) Unable to adhere to follow-up;
3) Surgical contraindications (including bleeding, coagulopathy, severe cardiopulmonary dysfunction);
4) Systemic therapy or local therapy (including chemotherapy, immunotherapy, targeted therapy with other drugs, radiofrequency ablation, radiotherapy, cryotherapy or vascular embolization) before medication;
5) Patients who have concomitant active malignant tumor diseases other than renal cell carcinoma or history of other malignant tumor diseases within 5 years (excluding basal cell carcinoma and cervical carcinoma in situ after active treatment);
6) Active gastrointestinal bleeding;
7) Malabsorption syndromes (e.g., celiac disease, cystic fibrosis, inflammatory bowel disease, systemic sclerosis, and carcinoid syndrome);
8) Patients with confirmed HIV or hepatitis B;
9) Seizure or brain metastasis, spinal cord compression or meningeal metastasis (carcinomatous meningitis);
10) Severe uncontrolled disease or active infection;
11) Any of the following within 12 months prior to receiving targeted agent: myocardial infarction, uncontrolled angina, coronary/peripheral artery bypass graft, symptomatic congestive heart failure, stroke, or transient ischemic attack. Deep vein thrombosis or pulmonary embolism within 6 months prior to receiving targeted drug therapy;
12) Failure to meet laboratory criteria for receiving drug therapy;
13) Currently taking or expected to take effective inhibitors of CYP3A4 (eg, grapefruit juice, verapamil, ketoconazole, miconazole, itraconazole, telithromycin, erythromycin, clarithromycin, indinavir, ritonavir, saquinavir, nelfinavir, nefazodone, lopinavir, atazanavir, amprenavir, fosamprenavir, delavirdine);
14) Current use or anticipated to need for potent CYP3A4 agonists, CYP3A4, CYP1A2 inducers (e.g., carbamazepine, dexamethasone, felbamate, omeprazole, phenobarbital, phenytoin sodium, phenobarbital, primidone, nevirapine, rifampin, rifabutin, and St.

John’s Wort);

15) Patients requiring long-term use of glucocorticoids or immunosuppressive agents;

16) Fertile subjects do not agree to take effective contraceptive measures during the study and within 3 months after the last dose.

3.5 Study endpoints and evaluation criteria

3.5.1 Primary endpoint:

Degradation rate of tumor thrombus: According to Mayo grading and 301 grading criteria, radiographic assessment was performed for enrolled patients before treatment and after 6 and 12 weeks of treatment to calculate the proportion of patients with decreased tumor thrombus grading.

3.5.2 Secondary endpoints:

Percentage change of tumor thrombus height and percentage change of tumor thrombus volume: The measurement basis of tumor thrombus height: calculate the distance from the highest point of the proximal end of the tumor thrombus to the midpoint of the renal vein opening, and the measurement basis of tumor thrombus volume: measure the coronal and sagittal maximum diameter of the tumor thrombus and the vertical length of the tumor thrombus according to imaging, and calculate the tumor thrombus volume = 1/2 coronal maximum diameter * sagittal maximum diameter * tumor thrombus height using the formula.

Percentage of change in surgical decision-making: Surgeon-reported surgical strategies were compared to actual performed surgical strategies based on the following three criteria. Reference standards: 1. Change from "open surgery" to "minimally invasive surgery"; 2. Change from more invasive, more complex surgical methods to less invasive, simpler surgical methods (such as reducing liver turning operation, hepatic vascular occlusion, avoiding cardiopulmonary bypass and thoracotomy); 3. Avoiding intraoperative surgical position change.

Objective response rate (ORR): According to Response Evaluation Criteria in Solid Tumors Version 1.1, CR + PR;

Disease control rate (DCR): CR + PR + SD according to Response Evaluation Criteria in Solid Tumors Version 1.1;

Progression-free survival (PFS): defined as the time from the start of drug therapy to radiological confirmation of progression prior to surgery or recurrence of disease

following surgical resection or death from any cause;

Overall survival (OS): defined as the time from the start of drug therapy to death from any cause.

Incidence of surgical complications: assessed according to Clavien-Dindo complication grading system.

Incidence of AEs and SAEs: incidence and severity of AEs and SAEs as judged by the National Cancer Institute Common Terminology Criteria for Adverse Events (NCI-CTCAE) 5.0.

3.5.3 Mayo and 301 grading evaluation criteria

Grade		301 Clinic	Mayo Clinic
0	0a	Confined within renal vein not exceeding superior mesenteric artery (left)	Confined within renal vein
	0b	Confined within renal vein but with tumor thrombus beyond superior mesenteric artery (left)	
I		Between renal vein opening and first porta hepatis	Invasion into the inferior vena cava, apex ⩽ 2 cm from the opening of the renal vein
II		Between first porta hepatis and second porta hepatis	Below hepatic vein level, apex > 2 cm from renal vein opening
III		Between the second porta hepatis and the diaphragm	Hepatic vein level and above, below diaphragm
IV	IVa	Above diaphragm to right atrium	Inferior vena cava above diaphragm
	IVb	Right atrium	

4. Study Drug

4.1 Drug Interventions

Axitinib: Patients started taking axitinib at an initial dose of 5 mg orally bid on Day 1 of Week 1 of the treatment cycle. Take the drug under fasting state or with food at an interval of about 12h, and continue to take the drug until the study drug treatment discontinuation criteria are met. Referring to section 4.2 for drug dose adjustment.

Toripalimab: 240 mg toripalimab administered on Day 1 of each 3-week dosing

cycle over at least 60 minutes as the first intravenous infusion. If the first infusion was well tolerated, the duration of the second infusion could be reduced to 30 minutes (+ 30 minutes). If the 30-minute infusion was also well tolerated by the subject, all subsequent infusions could be completed within 30 minutes (+ 30 minutes); if an infusion reaction occurred during the previous infusion, the subsequent infusion should be maintained for at least 60 minutes.

Each scheduled dose was calculated from the date of first dose. Delays (not caused by adverse events) generally do not exceed 3 days, but may exceed days in special cases (such as holidays, etc.) after the cause is indicated. If the delay exceeds 3 days, it is recommended to recalculate the next scheduled dose with the actual date of this dose, and the time window for subsequent doses remains 3 days.

4.2 Drug Dose Modifications

Medication modifications will be based on drug-related adverse events judged by the investigator. According to the investigator, if the adverse events experienced by the subject are related to certain drugs but not related to others, only the dose of some drugs related to toxicity may be adjusted, and only the treatment of some drugs related to toxicity may be interrupted/delayed/terminated. If it cannot be determined whether the adverse events are related to certain medications only, an overall adjustment of all study medications is required.

Dose adjustment should be based on the highest grade of adverse events in each treatment cycle, and the next cycle of treatment should be started after the corresponding adverse events recover to grade 1 or baseline (except for alopecia, grade 2 fatigue, and grade 2 or below endocrine system adverse reactions that can be controlled by hormone replacement therapy). If the subject only interrupts/delays the treatment of some drugs related to toxicity, the uninterrupted/delayed treatment of other drugs should still be planned. Once toxicity has essentially recovered, all medication treatments should proceed as planned.

4.2.1 Modification for Toripalimab Injection

Treatment was held due to toxicity and subjects could continue treatment at the original dose until toxicity improved. Regardless of immune-related adverse reactions, refer to Table 3 for treatment modifications when the investigator determines that an AE is related to toripalimab.

Table 3 Toripalimab Injection (JS001) Treatment Modification Regimen

Adverse Event	Severity (NCI CTCAE v5.0)	Treatment modification scheme
Pneumonia	Grade 2	Withhold until improved to Grade 0-1
	Grade 3-4 or recurrent Grade 2	Permanent discontinuation
Diarrhoea and colitis	Grade 2-3	Withhold until improved to Grade 0-1
	Grade 4	Permanent discontinuation
Hepatitis	Grade 2, AST or ALT 3-5 times upper limit of normal or total bilirubin 1.5-3 X ULN	Withhold until improved to Grade 0-1
	Grade 3-4, AST or ALT > 5X ULN, or total bilirubin > 3x ULN	Permanent discontinuation
Nephritis	Grade 2-3 blood creatinine increased	Withhold until improved to Grade 0-1
	Grade 4 blood creatinine increased	Permanent discontinuation
Endocrine disorders	Symptomatic grade 2-3 hypothyroidism,<br>Grade 2-3 hyperthyroidism,<br>Grade 2-3 hypophysitis,<br>2 Grade adrenal insufficiency<br>3 Grade I hyperglycemia or type I diabetes	Withhold until improved to Grade 0-1
	Grade 4 hypothyroidism,<br>Grade 4 hyperthyroidism,<br>Grade 4 hypophysitis,<br>Grade 3-4 adrenal insufficiency,<br>Grade 4 hyperglycemia or Type I diabetes	Permanent discontinuation

Cutaneous adverse reactions	Grade 3 rash<br>Grade 4 rash, Stevens-Johnson Syndrome (SJS) or toxic epidermal necrolysis (TEN)	Withhold until improved to Grade 0-1
Thrombocytopenia	Grade 3	Withhold until improved to Grade 0-1
	Grade 4	Permanent discontinuation
Other	Grade 3-4 blood amylase increased or lipase increased<br>Grade 2-3 pancreatitis<br>Grade 2 Myocarditis [a]<br>Grade 2-3 Initial Other Immune-related Adverse Reactions	Withhold until improved to Grade 0-1
	Grade 4 or any recurrent pancreatitis<br>Grade 3-4 myocarditis<br>Grade 3-4 encephalitis<br>Grade 4 Initial Other Immune-related Adverse Reactions	Permanent discontinuation
Recurrent or persistent adverse reactions	Recurrent Grade 3-4 (except endocrine disorders)<br>Grade 2-3 adverse reactions not improved to grade 0-1 within 12 weeks after last dose (except endocrine disorders)<br>Cortical consolidation within 12 weeks of last dose<br>Alcohol failed to decrease to ≤ 10 mg/day prednisone equivalent	Permanent discontinuation
Infusion reaction	Grade 2	Reduce the dripping speed or suspend the administration, and consider resuming the administration when the

		symptoms are relieved and close Observed
	Grade 3-4	MICARDIS PLUS must be discontinued immediately and treated symptomatically Physic

A: The safety of whether HUMIRA can be restarted after myocarditis is improved to grade 0-1 after treatment is not clear.

B: Abnormal laboratory findings of Grade 3/4 without clinical significance, and the administration should be continued at the investigator 's discretion.

4.2.2 Dose Modification for Axitinib

Axitinib was started at 5 mg bid. Axitinib was tolerated for at least 2 weeks without grade 2 or higher adverse reactions (NCI-CTCAE 5.0 criteria) and blood pressure was well controlled (≤ 150/90 mmHg), with dose escalation permitted. If dose reduction from 5 mg bid is required, the recommended dose is 3 mg bid. If a further dose reduction is required, the recommended dose is 2 mg bid. Axitinib may be resumed to the previous dose level if treatment-related toxicities and adverse reactions recover, are tolerated by the subject, or if adverse reactions do not recur.

Axitinib was permanently discontinued if the subject was unable to tolerate 2 mg bid. Axitinib treatment could be interrupted due to toxicity. If the drug is discontinued for more than 3 consecutive weeks, reassessment of imaging should be considered to determine whether discontinuation of treatment is required for surgical intervention.

Regimens for axitinib regimen modifications due to adverse reactions are presented in Table 4.

Table 4 Axitinib Treatment Modification Schedule

Adverse Event	Severity (NCI CTCAE v5.0)	Axitinib Dose Modification Schedule
Hypertension	Systolic BP ≤ 150 mmHg and/or diastolic BP ≤ 100 mmHg	Continue original dose, antihypertensive treatment can be started
	Adduction pressure > 150 mmHg but < 160 mmHg,	Start antihypertensive treatment or increase one antihypertensive drug while continuing the

	and/or relaxation pressure > 100 mmHg but<br>< 105 mmHg	original dose; if both antihypertensive drugs are still uncontrolled, then decrease one dose of axitinib
	Systolic blood pressure > 160 mmHg and/or diastolic blood pressure > 105 mmHg	Hold axitinib until BP < 150/100 mmHg, adjust Antihypertensive drugs;<br>Reduce dose by 1 dose when axitinib is restarted<br>Note: Blood pressure should be monitored following axitinib suspension, usually in<br>Blood pressure decreased back 1-2 days after discontinuation
	Recurrent hypertension following dose reduction	Reduce the dosage of axitinib by one level again<br>**Permanent Discontinuation**:<br>• Severe and persistent hypertension despite antihypertensive therapy and dose reduction;<br>• Hypertensive crisis;<br>• Temporary or permanent neurological dysfunction due to uncontrolled hypertension
Proteinuria	Strip - or 1 +	Continue original dose
	If dipstick > 1 +, test 24-hour urine protein or urine protein/creatinine ratio (UPC), pending results<br>Maintain original dose	
	Urine protein < 3g/24h or UPC < 3	Continue original dose
	Urine protein ≥ 3g/24h or UPC ≥ 3	Withhold until urine protein < 3g/24h and decrease by 1 dose upon rechallenge<br>**Permanent discontinuation:**<br>Axitinib down to 2 mg and urinary protein remains ≥ 3g/24h
Diarrhoea	Grade 1-2	Continue original dose
	Grade 3	Reduce one dose to continue<br>Refer to principles for management of nonhematologic toxicities

	Grade 4	Refer to principles for management of nonhematologic toxicities
Hemorrhage	Grade 1	Hemoptysis should be treated with interruption of study treatment and evaluation of potential causes, with recovery at the investigator 's discretion;<br>Other Grade 1 Bleeding Events, continue original dose and monitor clinically
	Grade 2	Pulmonary or gastrointestinal bleeding (except hemorrhoidal bleeding), permanently discontinued and followed per protocol;<br>For other Grade 2 bleeding, suspend the administration until recovered to ≤ Grade 1;<br>Reduce dose by 1 dose when axitinib is restarted
	Grade 3-4	Permanently discontinued and followed per protocol
ALT, AST, or bilirubin increased	Grade 1	Continue original dose
	Grade 2	Withhold until recovery to ≤ Grade 1 or baseline
	Grade 3 or 4	Withhold until recovery to ≤ Grade 1 or baseline,<br>Reduce dose by 1 dose reduction upon re-administration of TINIB
Hyperthyroidism	Grade 1-2	Continue original dose
	Grade 3	Asymptomatic or symptomatic manageable symptoms, as judged by the investigator<br>Discontinue original dose or downtitrate
	Grade 4	Withhold until recovery to ≤ Grade 1 or baseline,<br>Reduce dose by 1 dose reduction upon re-administration of TINIB
Hypothyroidism	All grades	Thyroid replacement started and axitinib continued
Renal failure or nephritis	Grade 1-2	Continue original dose
	Grade 3-4	Withhold until recovery to ≤ Grade 1 or baseline,

		Reduce dose by 1 dose reduction upon re-administration of TINIB
Non-hematological toxicities, laboratory abnormalities, and/or other suspected non-immune-related, axitinib-related drug toxicities	Grade 1-2	Continue original dose
	Grade 3-4	Symptomatic treatment of manageable Grade 3 toxicities or asymptomatic Grade 3/4 biochemical abnormalities (except liver function parameters), and the original dose may be continued at the investigator 's discretion;<br>Reduce the dose by 1 dose level for other Grade 3 toxicities to continue treatment;<br>Other Grade 4 non-hematological abnormalities, hold until recovered<br>≤ Grade 1 or baseline level;<br>Reduce axitinib dose by 1 dose decrement upon re-administration
	Grade 3-4 Discontinuation	**Permanent discontinuation:**<br>Recurrent serious or Grade 3 adverse reactions;<br>Any life-threatening adverse event;<br>Subjects experiencing the following events will be permanently discontinued: reversible posterior leukoencephalopathy syndrome (RPLS), arterial thrombosis/ischemia
Abnormal Hematology Laboratory Values	Grade 1-3	Continue original dose
	Grade 4	Withhold until recovery to ≤ Grade 2 or baseline and resume axitinib at 1 dose decrement<br>Note: Axitinib may be continued for Grade 4 lymphopenia not associated with a clinical event (eg, opportunistic infection)

4.3 Medication Compliance

Treatment with trial drug should be administered according to the dosage and method specified in the protocol. The date of administration of toripalimab, start and

stop time of infusion, and medication delay should be accurately recorded in the CRF. Subjects should record the information of medication/missed/wrong administration, blood pressure monitoring results and subjective discomfort symptoms of axitinib taken outside the hospital on the medication diary card. During each visit to the hospital, subjects will return the medication diary card last issued to the investigator. The investigator will make medication compliance judgment according to the medication conditions, perform medical judgment on abnormal blood pressure monitoring results and subjective discomfort symptoms, and record them in the original medical records.

4.4 Concomitant Therapy

Concomitant therapy is additional therapy administered at the investigator 's discretion based on consideration of the subject' s interest. All concomitant medications, blood products, and non-drug interventions (eg, punctures) received by the subject should be recorded in the CRF in strict accordance with GCP requirements from screening (28 days prior to first dose) through the end of the study safety visit. There was no prophylaxis with toripalimab. Other prophylactic medications should be administered according to standard of care.

In addition to the content mentioned above, subjects should refer to the latest version of the drug’s instructions or medical practice on the market for contraindications and precautions during treatment with the investigational drug.

5. Study Procedures

5.1 Screening Period

Written informed consent for participation in the study must be obtained prior to performing any study-specific screening tests or assessments, except for tumor imaging tests and tumor tissue sample collection within the specified timelines.

All screening assessments must be completed and results reviewed to confirm that patients meet all selection criteria before receiving study treatment. The investigator recorded information on all screened patients, screening tests and results, confirmed patient eligibility, or documented reasons for screening failure.

Abnormal screening results involving exclusion criteria are only allowed to be repeated once during the screening period, and the results closest to the first trial medication are used to decide whether a patient can be in the group. Subjects who have

previously failed screening will be allowed to be re-screened and the investigator will assess eligibility based on the most recent screening assessment. Re-sign the informed consent form and re-assign the screening number when re-sorting. Screening assessments: completed within 28 days prior to first trial drug if not otherwise specified.

[Signed informed consent] In addition to tumor imaging examination and tumor biopsy within the specified timelines, the informed consent form signed by the subject must be obtained before any study procedure is started.

[Demographic data] Including gender, date of birth, ethnicity, etc.

[Tumor diagnosis] Including clinical diagnosis, pathological diagnosis, date of diagnosis, clinical stage, histological type, pathological grade, primary tumor and metastatic lesion sites, tumor thrombus level, etc.

[Past tumor history and treatment history] Tumor history: including the first clinical diagnosis, pathological diagnosis and the date of the first diagnosis;

History of surgical treatment: History of surgery for primary lesion and/or metastatic lesion, including name of surgery, operation time, type of surgery (radical/palliative) and whether there is neoadjuvant therapy;

Drug treatment history (including neoadjuvant/adjuvant therapy): chemotherapy history, targeted drug treatment history, cytokine treatment history, etc., including medication regimen, drug name, medication time, dose, best efficacy and time to disease progression or recurrence after the last systemic treatment;

History of radiotherapy: including radiotherapy method, time, dose and site (systemic/local).

[Concomitant diseases] Concomitant diseases and relevant treatment history (such as diabetes, hypertension and other chronic diseases).

[Vital signs] should be measured after at least 5 minutes of comfortable rest and within 7 days before the first dose of trial medication.

[ECOG score] See Appendix for determination criteria, which should be completed within 7 days prior to the first dose of investigational product.

[Physical examination] Complete examination (including height, weight, etc.), which should be completed within 7 days before the first dose of study drug.

[Test items] Blood routine, urine routine, blood biochemistry, coagulation function, thyroid function test, pregnancy test and other test items should be completed within 7 days prior to the first dose.

[12-lead ECG] and [Echocardiography] completed within 14 days prior to the first dose of study drug.
[Infectious disease test] Completed within 28 days prior to the first dose of study drug.
[Tumor imaging examination] Including enhanced CT/MRI scans of chest, abdomen and pelvis, which were performed before signing the informed consent form and within 28 days before the first dose.
[Tumor tissue sample collection] For subjects participating in biomarker exploratory study, previous or fresh tumor tissue samples (capable of producing 6 slides) will be collected and completed within 28 days prior to the first dose of study drug.

5.2 Treatment Period

The treatment period starts from the first day of study drug administration until completion of surgery or study treatment. Screened eligible subjects entered the treatment period. Subsequent visits and imaging scheduled dates were calculated based on the date of the first trial medication, with a window of ± 3 days for each modality during the treatment period and ± 7 days for tumor imaging.

Information on medication/missed/wrong administration, blood pressure monitoring results and subjective discomfort symptoms of axitinib taken outside the hospital should be recorded by the subject on the medication diary card. At each visit to the hospital, the subject should hand back the last dispensed medication diary card to the investigator for medication compliance and safety judgment by the investigator.

On-treatment assessment items:

[Vital signs] Vital signs will be examined in each dosing cycle in the axitinib group within 30 minutes before each infusion and within 60 minutes after the completion of each infusion in the toripalimab group; blood pressure should be monitored every for subjects taking axitinib outside the hospital, and measurements will be recorded in the dosing diary card.
[Physical examination] Only targeted physical examination will be performed during treatment (after informed consent is signed, those who have been examined within 7 days before the first trial medication do not have to repeat C1D1).
[ECOG score] [blood routine] [urine routine] [blood biochemistry] [coagulation function] and other laboratory tests were performed at weeks 3, 7 and 12 after treatment. Tests were performed after initiation of study drug in the sunitinib arm. In case of

suspension or dose adjustment due to hematological toxicity, it is recommended to reexamine blood routine every week until recovery. If neutrophil ≤ 1.0 × 10^9/L or platelet ≤ 50 × 10^9/L, frequency of re-examination should be increased (recommended once every 2-3 days). Increased testing frequency is required for ALT or AST elevations of 2-fold or 2-fold from baseline abnormalities (recommended 1-2 times/week).

[Thyroid function tests] Tests were performed after starting study drug treatment (with the same frequency as urine routine), at the end-of-treatment visit, and at safety follow-up in the toripalimab combined with axitinib and sunitinib groups. If clinically significant changes in thyroid function occur, prompt management is required and consultation with endocrinology is recommended and pituitary function tests are performed as needed.

[12-lead ECG] Performed after initiation of study drug (the frequency of examination is the same as urine routine), and added when clinically indicated.

[Tumor imaging examination] Including chest, abdomen, pelvic cavity and sites with lesions, using the same method as the baseline, and preferably assessed by the same investigator or radiologist. Imaging tests and efficacy evaluation were performed at week 7 after the start of treatment and before surgery, and efficacy was assessed according to RECIST 1.1 criteria.

[Adverse Events] [Concomitant Medications] Continuously monitor and record.

5.3 Surgical Intervention

For patients who meet the surgical indications as assessed by the investigator, inferior vena cava tumor thrombectomy combined with radical nephrectomy is performed, and the following patient information needs to be collected perioperatively.

[Drug efficacy] Changes in the maximum diameter of the primary tumor, changes in the height and maximum diameter of the tumor thrombus, and changes in the number and maximum diameter of metastases (according to the patient's CT/MRI before treatment and before surgery, with reference to the Response Evaluation Criteria for Solid Tumors Version 1.1);

[Operation-related data] Surgical procedures, operation time, intraoperative blood loss, perioperative complications, hospital stays, drainage tube removal time;

[Pathological results] Postoperative pathological type, pathological stage, grade,

pathological response rate and so on.

5.4 Postoperative follow-up period

Patients were followed up for at least 1 year after surgery, every 3 months for 1 year, and every 3 months for telephone follow-up or visits, for a total follow-up frequency of approximately 4 times/year. The following data were collected during follow-up.

[ECOG score] See Appendix for determination criteria.

[Test items] Blood routine, urine routine, blood biochemistry, coagulation function, thyroid function test and other test items.

[Tumor imaging examination] Including chest, abdomen, pelvic cavity and sites with lesions, postoperative survival was assessed according to RECIST 1.1 criteria using the same method as baseline.

5.5 Sample Collection and Testing

Corresponding biological samples were collected at different treatment time nodes. Including pathological puncture specimens obtained before treatment, tumor thrombus, primary tumor lesions and paratumoral tissues collected after surgery, one of the above fresh tissue samples was submitted for transcriptome sequencing, one was cryopreserved in liquid nitrogen for subsequent detection, and the remaining tissues were embedded in paraffin to make paraffin blocks for retention.

6. Sample Size Estimation

Simon-2 stage method was used to calculate the sample size required for this trial. According to previously reported data, this clinical trial was designed based on the hypothesis that combined targeted immunotherapy would have limited efficacy for inferior vena cava tumor thrombus if the proportion of patients with highly degraded tumor thrombus was ≤ 10% (H0). In contrast, the proportion of patients with highly degraded tumor thrombus was > 30% (H1) after receiving drug treatment, and the combined treatment regimen was considered to have a significant effect on inferior vena cava tumor thrombus and suitable for further clinical studies. Type 1 error is set to 0.1 and power is set to 0.9. In this study, a two-stage design was used to include 25 patients, including 16 and 9 patients in the first and second stages, respectively.

Following the first stage of the trial, if ≤ 1 patient responded (tumor thrombus downgraded), the null hypothesis was accepted and the trial was terminated. If ≥ 2 patients responded (tumor thrombus downgraded), they entered Stage II. After completion of both phases of the trial, the null hypothesis was rejected if ≥ 5 patients responded to treatment.

7. Flow Chart

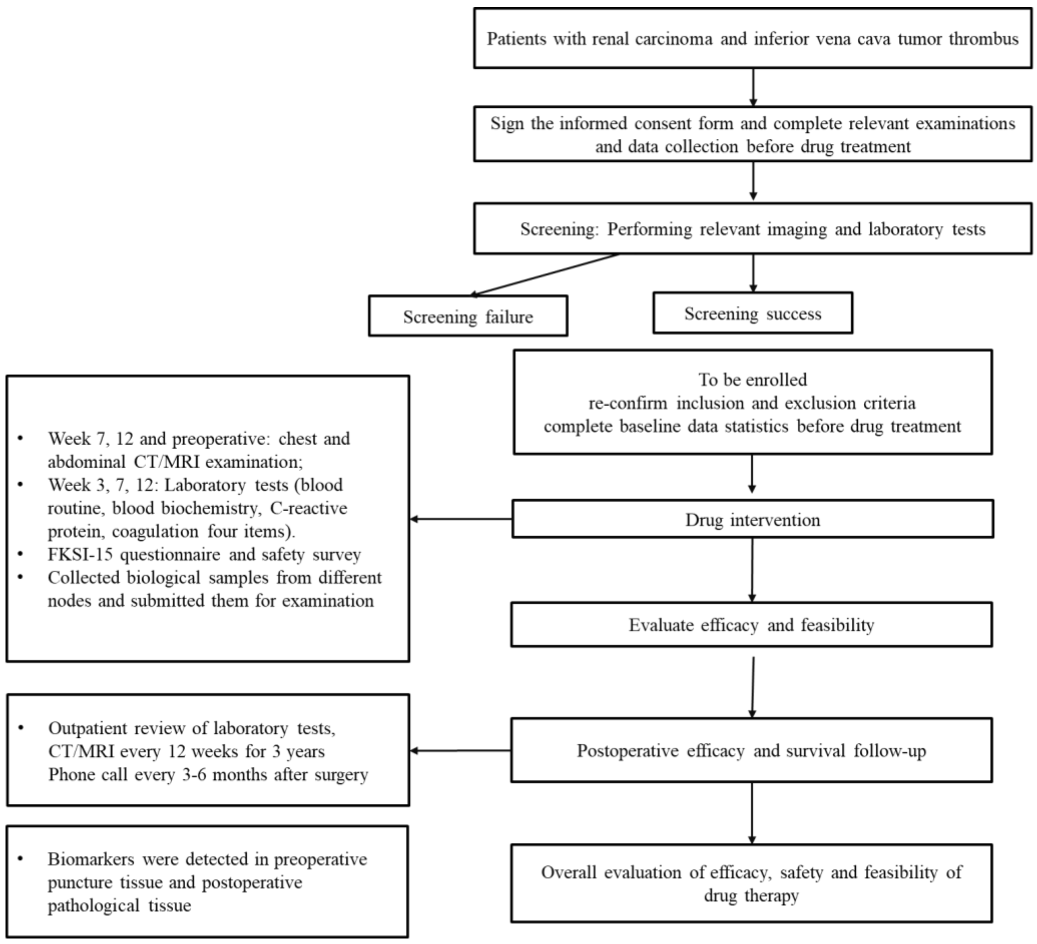

8. Management and Handling Measures of Adverse Events

8.1 Definition of Adverse Events

An adverse event refers to an untoward medical occurrence in a clinical trial subject administered a medicinal product and which does not necessarily have a causal relationship with this treatment. An adverse event can be any unfavorable and unintended symptom, sign, laboratory abnormality, or disease, including the following:

1) Exacerbation of pre-existing (before entering the clinical trial) medical

conditions/diseases (including aggravation of symptoms, signs, laboratory abnormalities; except for events clearly related to disease progression);

2) Any new adverse event: any new adverse medical condition (including symptoms, signs and newly diagnosed diseases);

3) Abnormal clinically significant laboratory values or results that are not due to concomitant disease.

Investigators should record in detail any adverse events occurred in subjects, including: description of adverse events and all related symptoms, occurrence time, severity, cause of adverse events, correlation with the investigational product, duration, measures taken, final results and outcome.

The name of the adverse event should be recorded on the CRF with the name of the diagnosis or disease. If the name of the adverse event can't be defined or the investigator considers that the diagnosis or disease name can't be used as the name of the adverse event, the clinical condition or symptom will be recorded as the name of the adverse event on the CRF.

8.2 Definition of Serious Adverse Events

A serious adverse event refers to an untoward medical occurrence that meets one or more of the following criteria:

- Events leading to death;
- A life-threatening event (defined as an event in which the subject was at immediate risk of death at the time of the event);
- Events requiring hospitalization or prolongation of hospitalization;
- An event that results in persistent or significant disability/incapacity/incapacity;
- Congenital anomaly or birth defect;

Other important medical events (defined as those events that, although not immediately life-threatening, result in death or hospitalization, may jeopardize the subject or may require intervention [such as medical or surgical] to prevent serious consequences listed in the definition above) based on appropriate medical and scientific judgment. Examples of such events include, but are not limited to, allergic

bronchospasm requiring intensive treatment in the emergency room or at home, hematologic cachexia or convulsions not requiring hospitalization, potential drug-induced liver injury, suspected transmission of a pathogen (eg, pathogenic or nonpathogenic) via study drug, secondary neoplasm, etc.

8.3 Recording and Reporting of AEs/SAEs

Only SAEs are recorded and reported from the time the patient signed informed consent until the start of study medication. Following initiation of the study, all AEs and SAEs, regardless of relationship to study drug, will be reported until 30 days after the last dose of study treatment or until initiation of a new anticancer therapy, whichever occurs first. In the toripalimab plus axitinib arm, SAEs were reported until 30 to 90 days after the last study dose (30 days after the last dose of axitinib if the subject had discontinued toripalimab and received axitinib only), or the start of a new anticancer therapy, whichever came first. Any SAEs that occur at any time after treatment discontinuation and is assessed as related to toripalimab should be reported by the investigator. In the sunitinib arm, all AEs (regardless of relationship to study drug) were recorded and all SAEs were reported from the start of study drug through 30 days after the last dose of study drug.

For all AEs occurring during the clinical study, whether or not related to the study drug, the following measures should be taken:

1) The investigator should immediately take appropriate protective measures to ensure the safety of the subjects, and experts should make a diagnosis and state the reasons;

2) If the study is terminated, the investigator should also regularly examine the subjects and fill in the date of termination (the date when the investigational product is stopped), the reason for termination and the detailed process in the CRF;

3) The investigator should follow up all AEs until any of the following occurs:

- AE is relieved or improved to baseline or better;
- The investigator confirms that the event is in a stable state and no further improvement is expected;

- Death of the subject;
- The subject has lost contact or withdrawn informed consent;
- Investigator confirms AE is unrelated to study treatment;
- Subjects start new anti-tumor therapy.

8.4 Symptomatic treatment and recommendations for common adverse reactions

8.4.1 Immuno-oncology Drug Safety Management Rules

Immuno-oncology (I-O) drug-induced adverse events differ from other types of anti-tumor drugs, with their particularities in severity and duration. Toripalimab belongs to this class of drugs and therefore requires early recognition and treatment of adverse events caused by it to reduce the occurrence of serious toxic events. Specific measures please refer to the Investigator's Brochure for the management of immune-related adverse events (irAEs) and other special adverse events.

8.4.2 Recommendations for the management of common adverse reactions of targeted therapy

a) Hypertension: Before initiation of targeted drug therapy, ensure that blood pressure is well controlled, and inform patients that blood pressure should be measured regularly, antihypertensive drugs should be taken according to the doctors' advice, and danger signs caused by hypertension should be recognized, such as blurred vision, headache, and palpitations. Treatment varies according to the degree of blood pressure elevation after the start of treatment. During treatment, if systolic blood pressure was > 150 mmHg or diastolic blood pressure was > 100 mmHg, the intensity of treatment was increased by changing or adding antihypertensive drugs, and axitinib was reduced by 1 dose level if the maximum intensity of antihypertensive treatment had been reached. If systolic blood pressure > 160 mmHg or diastolic blood pressure > 105 mmHg, the targeted drug therapy will be suspended, the antihypertensive drug will be adjusted, and the treatment will be resumed and the dose level will be reduced by 1 dose level after the blood pressure is < 150/100 mmHg. If increased blood pressure (> 150/100 mmHg) recurred after dose reduction, dose reduction continued by 1 dose level.

b) Fatigue: refers broadly to a series of discomfort such as tiredness, tiredness, or physical decline. Symptomatic patients should be initially assessed for hemoglobin, thyroid function, nutritional group status, and state of consciousness, and closely monitored for treatment-related anorexia and cachexia. Treat the cause of fatigue and encourage the patient to increase activity and nutritional counseling. Remind patients to promptly seek medical attention to oncology once fatigue has worsened.

c) Diarrhea: Evaluate patients' bowel habits at baseline and investigate whether they are caused by concomitant medications. For diarrhea Grade 1 and 2, diet modification, oral rehydration and antidiarrheal medication were administered. For Grade 3 and 4 patients, treatment was actively withheld or dose reduced, outpatient intravenous fluids were administered, and antidiarrheal medications were administered. Especially for Grade 4 patients, inpatient fluid replacement was recommended to allow adequate rest of the gastrointestinal tract.

d) Hand-foot syndrome: characterized by redness of the skin on the palms or soles of the feet. Obvious discomfort, swelling, tingling, etc. For Grade 1 patients, detect changes in disease, avoid contact with overheated water, avoid tight shoes and socks, and use moisturizing creams and keratolytic agents. For Grade 2 patients, topical analgesics were added to Grade 1 management to relieve symptoms, and clobetasol ointment was applied twice daily. For Grade 3 patients, urea cream and lidocaine cream were added.

e) Nausea and vomiting: Patients in Grade 1 and 2 maintained the original dose of the drug, adjusted diet, antiemetic treatment, and active intravenous fluid replacement for patients in Grade 2. Patients in Grade 3 and 4 will have the targeted drug dose reduced or suspended, and hospitalization for active intravenous fluid replacement and symptomatic treatment is recommended.

f) Gastrointestinal bleeding: gastrointestinal bleeding, including fecal occult blood (+ +) or more, hematemesis or bloody stool, etc., should be actively treated symptomatically. Patients with upper gastrointestinal bleeding should be fasted, and given acid suppression, protection of gastric mucosa, hemostasis (tranexamic acid, reptilase, etc.), octreotide can be used if necessary; patients with lower gastrointestinal bleeding should be given hemostasis, blood transfusion and

supportive treatment; patients with uncontrolled bleeding should be treated with surgical assistance.

g) See package insert for other adverse reaction management measures.

9. Quality Control Measures

9.1 Ethics

This study will be conducted in accordance with the Declaration of Helsinki and GCP. The experimental design protocol will be submitted to the Medical Committee of the Chinese People's Liberation Army. The experimental design protocol will be submitted to the Ethics Committee of the Chinese People's Liberation Army Medical College for review and guidance and supervision to protect the dignity, safety and rights and interests of subjects. This study protocol can only be implemented after being approved by the Ethics Committee of the participating institution. The investigator will ensure that this clinical study is conducted in accordance with the laws, regulations, scientific and ethical standards for medical research of the People's Republic of China. In case of any amendment to the study protocol during the study, the study protocol and informed consent form shall be revised in writing and submitted to the Ethics Committee for review and approval, and the consent of the subject shall be obtained again. For patients who do not meet the inclusion criteria or drop out of the trial, a specialist will be arranged to perform an outpatient review or telephone follow-up, and instruct and arrange for them to undergo surgical treatment or continue targeted drug therapy.

9.2 Informed Consent

Before the start of the study, the investigator should introduce the purpose, procedures, methods and possible risks of the study to the subjects in a complete and comprehensive manner, and should allow each subject to clearly understand their rights, risks and benefits to be burned. Each subject participating in this clinical study can be enrolled and started the study only after obtaining their consent and signing.

9.3 Confidentiality

During the study period, the patient 's personal and research data will be replaced

by codes or numbers, and strictly kept confidential. Only the relevant doctors can obtain and contact the above data, and the patients' privacy right will be well protected. Personal data will not be involved in the publication of the study results.

10. Risk and Benefit Assessment

10.1 Benefit (personal and social interests)

For patients with renal cell carcinoma and inferior vena cava tumor thrombus, preoperative targeted combined with immune drug therapy is expected to reduce the height of tumor thrombus and reduce the size of primary tumor, thereby reducing the stage of tumor and surgical difficulty and enhancing the safety and feasibility of surgical treatment, especially for patients with level III or higher tumor thrombus, if successful downgrading can avoid thoracotomy and cardiopulmonary bypass, which is expected to reduce the incidence of perioperative fatal complications while reducing the difficulty of surgery.

At the same time, analyzing the relationship between the efficacy of targeted combined immunotherapy and the clinical data and characteristics of patients is helpful to select renal cell carcinoma patients sensitive to combined therapy, so as to improve the effect of targeted/immunotherapy and reduce the medical expenditure of patients and social medical costs.

10.2 Risks

Patients are at risk of thrombus/tumor thrombus shedding during preoperative targeted combined immune drug therapy, especially for patients with tumor thrombus combined with thrombus in the proximal inferior vena cava, strict bed rest should be performed to avoid exertion or strenuous exercise for such patients. Prophylactic use of anticoagulants may reduce the occurrence of thrombosis-related events, while coagulation parameters need to be measured in patients to avoid increasing the risk of bleeding.

Adverse reactions are another major concern in combination drug therapy. Axitinib, as a new drug marketed at a later stage, has a reduced severity of adverse reactions compared with sorafenib. Common adverse reactions include diarrhea, hypertension, fatigue, hand-foot syndrome, liver function damage, etc., most of which are mild, and CTCAE grade is mainly 1-2. For adverse reactions, relevant personnel

will regularly follow up and monitor, remind patients to always pay attention to their own reactions, and regularly review according to the doctors' guidance. Once discomfort occurs, timely visit relevant departments to prevent the occurrence of serious adverse reactions and avoid affecting the subsequent surgical treatment.

Radical nephrectomy combined with removal of vena cava tumor thrombus is one of the operations with very high risk in urology, which often faces complications such as massive hemorrhage and thrombosis detachment and death. Targeted drug therapy may also increase the risk of intraoperative bleeding and incision-related complications. Tests of wound neovascularization have shown that anti-VEGF agents cause delayed wound healing and therefore require a period of discontinuation before surgery, and literature reports have shown that although the risk of incision-related complications is high, the overall incidence of surgical complications and serious complications have not increased.

11. Feasibility Analysis

Preliminary work basis: We retrospectively analyzed the clinical data of patients with renal cell carcinoma and inferior vena cava tumor thrombus who visited our hospital, and selected 14 patients who received preoperative targeted therapy, 8 patients received sunitinib, 5 patients received sorafenib, and 1 patient received axitinib, with a median treatment time of 2 cycles (1-6 cycles), 4 patients had significantly shortened tumor thrombus length after targeted therapy, 10 patients had no significant change in tumor thrombus length, with a median change in tumor thrombus height of -0.4 cm (-4.23 to 0.17 cm) and a median change in the maximum diameter of -0.2 cm (-1.57 to 0.8 cm). The tumor thrombus grade of 2 patients was reduced from grade III to grade II, and both patients were treated with robotic surgery, which avoided the second porta hepatis blocking and robotic left and right hepatic inversion during the operation and reduced the difficulty of surgery. All adverse reactions were Grade 1-2, and no patient had intermittent discontinuation or dose reduction. Previous preliminary experiments showed that patients with renal cell carcinoma and tumor thrombus tolerated immunotherapeutic drugs well.

Working conditions: With the help of our first-class medical and nursing team and research team, advanced da Vinci robotic system and laparoscopic surgery system, 211 patients with renal cell carcinoma and inferior vena cava tumor thrombus have been

treated in our department since 2006, with incomparable high-quality patient volume in other domestic hospitals, which is conducive to carrying out prospective studies.

References

1. Psutka, S.P. and B.C. Leibovich, Management of inferior vena cava tumor thrombus in locally advanced renal cell carcinoma. Therapeutic Advances in Urology, 2015. **7**(4): p. 216.
2. Bex, A., et al., Progression of a caval vein thrombus in two patients with primary renal cell carcinoma on pretreatment with sunitinib. Acta Oncologica, 2010. 49(4): p. 520.
3. Thomas, A.A., et al., Surgical resection of renal cell carcinoma after targeted therapy. J Urol, 2009. 182(3): p. 881-886.
4. Rini, B.I., et al., The effect of sunitinib on primary renal cell carcinoma and facilitation of subsequent surgery. Journal of Urology, 2012. 187(5): p. 1548.
5. Shuch, B., et al., Neoadjuvant targeted therapy and advanced kidney cancer: observations and implications for a new treatment paradigm. Bju International, 2008. 102(6): p. 692–696.
6. Rixe, O., et al., Axitinib treatment in patients with cytokine-refractory metastatic renal-cell cancer: a phase II study. Lancet Oncology, 2007. 8(11): p. 975-984.
7. Motzer RJ, Escudier B, McDermott DF, et al. Nivolumab versus Everolimus in Advanced Renal-Cell Carcinoma[J]. N Engl J Med, 2015, 373(19):1803-1813.
8. Rini BI, Escudier B, Tomczak P, Kaprin A, Szczylik C, Hutson TE, et al. Effectiveness of axitinib versus sorafenib in advanced renal cell carcinoma (AXIS): a Comparative randomised phase 3 trial [J]. Lancet. 2011; 378:1931-9.
9. Motzer RJ, Escudier B, Tomczak P, Hutson TE, Michaelson MD, Negrier S, et al. Axitinib versus sorafenib as second-line treatment for advanced renal cell carcinoma: overall survival analysis and updated results from a randomised renal phase 3 trial [J]. Lancet Oncol. 2013 May 14 (6): 552-62.
10. Hutson TE, Lesovoy V, Al-Shukri S, Stus V, Lipatov ON, Bair AH, et al. Axitinib versus sorafenib as open-label first-line therapy in patients with metastatic renal-cell carcinoma: a randomized phase 3 trial [J]. Lancet. 2013; 14:1287-94.
11. Motzer RJ, Jonasch E, Agarwal N, Beard C, Bhayani S, Chang S, et al. NCCN clinical practice guidelines in oncology (NCCN Guidelines ®. Kidney Cancer. Version 2.2016.

APPENDICES

Appendix 1: ECOG Performance Status Scoring Criteria

Score	Activity Level
0	Normal activity. Fully active, able to carry on all pre-disease performance without restriction.
1	Symptoms, but ambulatory. Restricted in physically strenuous activity, but ambulatory and able to carry out work of a light or sedentary nature (e.g., light housework, office work).
2	In bed <50% of the time. Ambulatory and capable of all self-care, but unable to carry out any work activities. Up and about more than 50% of waking hours.
3	In bed >50% of the time. Capable of only limited self-care, confined to bed or chair more than 50% of waking hours.
4	100% bedridden. Completely disabled. Cannot carry on any self-care. Totally confined to bed or chair.
5	Dead
